# A lesion-selective albumin-CTLA4Ig as a safe and effective treatment for collagen-induced arthritis

**DOI:** 10.1186/s41232-023-00264-8

**Published:** 2023-02-16

**Authors:** Fu-Yao Jiang, Yan-Zhu Zhang, Yuan-Hong Tai, Chien-Yu Chou, Yu-Ching Hsieh, Ya-Chi Chang, Hsiao-Chen Huang, Zhi-Qin Li, Yuan-Chin Hsieh, I-Ju Chen, Bo-Cheng Huang, Yu-Cheng Su, Wen-Wei Lin, Hsin-Chieh Lin, Jui-I Chao, Shyng-Shiou F. Yuan, Yun-Ming Wang, Tian-Lu Cheng, Shey-Cherng Tzou

**Affiliations:** 1 Department of Biological Science and Technology, National Yang Ming Chiao Tung University, Hsinchu, Taiwan, Republic of China; 2 Institute of Molecular Medicine and Bioengineering, National Yang Ming Chiao Tung University, Hsinchu, Taiwan, Republic of China; 3 School of Medicine for International Students, I-Shou University, Kaoshiung, Taiwan, Republic of China; 4 School of Medicine, I-Shou University, Kaohsiung, Taiwan, Republic of China; 5 Institute of Biomedical Sciences, National Sun Yat-Sen University, Kaohsiung, Taiwan, Republic of China; 6 Drug Development and Value Creation Research Center, Kaohsiung Medical University, Kaohsiung, Taiwan, Republic of China; 7 Department of Laboratory Medicine, Post Baccalaureate Medicine, College of Medicine, Kaohsiung Medical University, Kaohsiung, Taiwan, Republic of China; 8 Department of Materials Science and Engineering, National Yang Ming Chiao Tung University, Hsinchu, Taiwan, Republic of China; 9 Translational Research Center, Department of Obstetrics and Gynecology, Kaohsiung Medical University Hospital, and Faculty and College of Medicine, Kaohsiung Medical University, Kaohsiung, Taiwan, Republic of China; 10 Department of Biomedical Science and Environmental Biology, Kaohsiung Medical University, Kaohsiung, Taiwan, Republic of China; 11 Graduate Institute of Medicine, Kaohsiung Medical University, Kaohsiung, Taiwan, Republic of China; 12 Center for Intelligent Drug Systems and Smart Bio-devices (IDS2B), National Yang Ming Chiao Tung University, Hsinchu, Taiwan, Republic of China

**Keywords:** Albumin, CTLA4Ig, Adverse effects, Inflammatory lesion-selective, Protein engineering, Matrix metalloproteinase (MMP), Collagen-induced arthritis

## Abstract

**Background:**

CTLA4Ig is a dimeric fusion protein of the extracellular domain of cytotoxic T-lymphocyte protein 4 (CTLA4) and an Fc (Ig) fragment of human IgG_1_ that is approved for treating rheumatoid arthritis. However, CTLA4Ig may induce adverse effects. Developing a lesion-selective variant of CTLA4Ig may improve safety while maintaining the efficacy of the treatment.

**Methods:**

We linked albumin to the N-terminus of CTLA4Ig (termed Alb-CTLA4Ig) via a substrate sequence of matrix metalloproteinase (MMP). The binding activities and the biological activities of Alb-CTLA4Ig before and after MMP digestion were analyzed by a cell-based ELISA and an in vitro Jurkat T cell activation assay. The efficacy and safety of Alb-CTLA4Ig in treating joint inflammation were tested in mouse collagen-induced arthritis.

**Results:**

Alb-CTLA4Ig is stable and inactive under physiological conditions but can be fully activated by MMPs. The binding activity of nondigested Alb-CTLA4Ig was at least 10,000-fold weaker than that of MMP-digested Alb-CTLA4Ig. Nondigested Alb-CTLA4Ig was unable to inhibit Jurkat T cell activation, whereas MMP-digested Alb-CTLA4Ig was as potent as conventional CTLA4Ig in inhibiting the T cells. Alb-CTLA4Ig was converted to CTLA4Ig in the inflamed joints to treat mouse collagen-induced arthritis, showing similar efficacy to that of conventional CTLA4Ig. In contrast to conventional CTLA4Ig, Alb-CTLA4Ig did not inhibit the antimicrobial responses in the spleens of the treated mice.

**Conclusions:**

Our study indicates that Alb-CTLA4Ig can be activated by MMPs to suppress tissue inflammation in situ. Thus, Alb-CTLA4Ig is a safe and effective treatment for collagen-induced arthritis in mice.

**Supplementary Information:**

The online version contains supplementary material available at 10.1186/s41232-023-00264-8.

## Background

Chronic inflammatory diseases such as autoimmune diseases are major health issues globally. Many chronic inflammatory diseases are characterized by dysregulated T cell activation. For example, arthritogenic T cells are thought to be activated in the lymph nodes and then enter the joints of rheumatoid arthritis. Activated T cells recruit an array of inflammatory cells that ultimately cause joint destruction [1, 2]. Thus, the activation of pathogenic T cells plays a central role in many chronic inflammatory diseases. Accordingly, inhibiting T cells with CTLA4Ig or anti-CD3 antibodies is a promising therapeutic strategy for chronic inflammatory diseases.

CTLA4Ig (abatacept) is a dimeric fusion protein of the extracellular domain (ECD) of cytotoxic T-lymphocyte protein 4 (CTLA4) and an Fc (Ig) fragment of human IgG_1_. CTLA4Ig has been approved by the FDA for treating rheumatoid arthritis [3, 4]. CTLA4Ig binds to CD80/CD86 with a stronger affinity than CD28 [5] and thus efficiently blocks the CD28 signaling required for full T cell activation [6]. However, CTLA4Ig may induce adverse effects such as infections in treated patients [7]. The use of belatacept, a second-generation CTLA4Ig that differs from the original form by two amino acids, is associated with a high incidence of post-transplant lymphoproliferative disorder in the subsets of kidney transplantation patients [8]. As a result, patients may discontinue CTLA4Ig therapy due to severe adverse effects [9, 10]. Furthermore, CTLA4Ig is associated with an increased incidence of skin cancers [11, 12], raising the concern that long-term systemic suppression of T cells by this agent may compromise cancer immunosurveillance. These adverse effects are most likely due to a nondiscriminatory action of CTLA4Ig.

One potential solution to this dilemma may lie in the selective inhibition of inflammation in the lesion sites. In addition, lesion-selective activation of therapeutic agents prevents premature binding of the therapeutic agents to ligands expressed in nondiseased tissues (antigen sink effect [13]). It is well known that inflammatory lesions are characterized by the overexpression of matrix metalloproteinases (MMPs) [14, 15]. These MMPs can serve as biomarkers for diseases [16, 17] and are active players in disease progression [18, 19]. Accordingly, we have also developed several reagents that exploit MMPs to activate therapeutic effects or imaging activity [20–22]. Therefore, MMPs in inflammatory tissues are rational targets for designing lesion-selective therapeutic agents. However, while most protein engineering methods have been developed for constructing lesion-selective antibodies [23, 24], methods designed for other therapeutic proteins, such as CTLA4Ig, are relatively scarce.

**Fig. 1:**
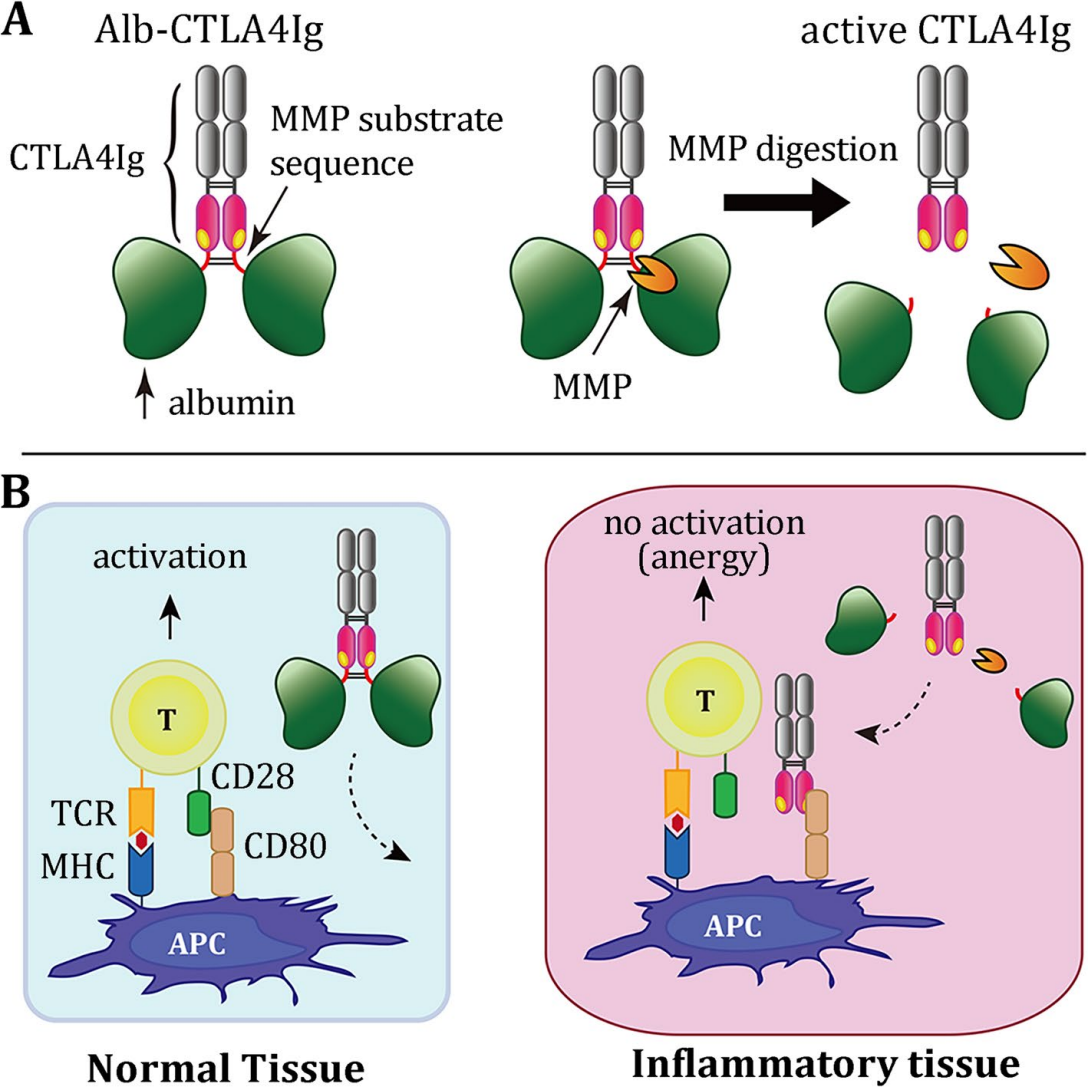
The strategy to develop a lesion-selective Alb-CTLA-4Ig for the treatment of collagen-induced arthritis. **A** Albumin is linked (via a hinge sequence) to the N-terminus of CTLA4Ig to mask its binding sites to CD80. Albumin can be removed by MMP digestion at the hinge sequence to restore the binding activity. **B** When Alb-CTLA4Ig circulates through normal tissues (left), it is inactive due to the low expression of MMPs. However, Alb-CTLA4Ig is converted to an active form (CTLA4Ig) when it enters the inflammatory tissues where MMPs are selectively overexpressed. The activated form can bind to CD80 or CD86 to block the second signal required for full T cell activation.

To attain such lesion-selective inhibition of inflammation, we developed a novel pro-CTLA4Ig fusion protein by linking albumin, via a hinge sequence of immunoglobulins, to the N-terminus of CTLA4Ig (termed Alb-CTLA4Ig hereafter) to sterically block the binding sites of CTLA4Ig. Several biological-chemical properties of albumin render it a favorable masking domain, including its relatively large molecular size (to cover sufficient space over CTLA4Ig), excellent solubility, and long half-life in the serum [25]. Alb-CTLA4Ig is designed for cleavage by MMPs overexpressed in inflammatory tissues to restore its binding activity (Fig. 1). We analyzed the stability, binding activity, and biological activity of Alb-CTLA4Ig in vitro and its activation/conversion and treatment efficacy for collagen-induced arthritis in vivo. Finally, we tested whether Alb-CTLA4Ig induced fewer adverse effects in vivo. Our results indicate that Alb-CTLA4Ig may be selectively activated to suppress joint inflammation but does not provoke systemic immune suppression, achieving a safe and effective treatment for collagen-induced arthritis in mice.

## Methods

### Expression and purification of Alb-CTLA4Ig

ExpiCHO cells (Thermo Fisher Scientific, Waltham, MA, USA) were transfected with CTLA4Ig and Alb-CTLA4Ig expression vectors [see Additional file 2: Supplementary methods for the construction of mouse CTLA4Ig (mCTLA4Ig), mouse Alb-CTLA4Ig (mAlb-CTLA4Ig), human CTLA4Ig (hCTLA4Ig), and human Alb-CTLA4Ig (hAlb-CTLA4Ig)] using the transfection reagents provided in the kit. mCTLA4Ig, mAlb-CTLA4Ig, hCTLA4Ig, and hAlb-CTLA4Ig in the culture supernatants of the ExpiCHO transfectants were purified by a HisTrap column (GE Life Sciences, Malborough, MA, USA) using a previously described procedure [26]. Purified proteins were dialyzed in Tris-buffered saline (50 mM Tris, pH 7.5, 150 mM NaCl) for subsequent tests or stored at − 80 °C until use.

### Digestion of Alb-CTLA4Ig

MMP2/9 are type IV collagenases; thus, we routinely used type IV collagenase (Sigma-Aldrich) to digest the recombinant proteins for in vitro assays. Following a previously established study [27], mAlb-CTLA4Ig (300 ng per reaction) was digested by 2 μg (unless indicated otherwise) of type IV collagenase in 40 μl in TCNB buffer (50 mM Tris, 150 mM NaCl, 10 mM CaCl_2_, 0.05% Brij-35, pH 7.5) containing 0.1% BSA at 37 °C for 1 h. In addition to type IV collagenase, hAlb-CTLA4Ig (300 ng per reaction) was tested for cleavage by the indicated amounts of recombinant human MMP3 (Abcam, Cambridge, MA, USA) in TCNB buffer 0.1% BSA. Digestion was stopped by adding EDTA to 10 mM. Digested proteins were separated by reducing SDS-PAGE and analyzed by western blotting using anti-mouse or anti-human IgG Fcγ antibodies (both from Jackson ImmunoResearch Laboratories, West Grove, PA, USA) or anti-mouse or anti-human CTLA4 antibodies (R&D Systems, Minneapolis, MN, USA) to evaluate the extent of digestion used in binding assays or biological activity.

To assess whether sufficient MMPs are expressed in inflammatory tissues to digest mAlb-CTLA4Ig, we aspirated synovial fluid lavages after injecting 10 μl (5 μl, twice) of DMEM into the knee joints of mice that developed collagen-induced arthritis (CIA). mAlb-CTLA4Ig (300 ng) was incubated in CIA synovial lavage fluid at 37 °C for 3 h. The degree of digestion was analyzed by reducing SDS-PAGE and western blotting using an anti-mouse CTLA4 antibody. To test whether mAlb-CTLA4Ig could be digested in the inflamed joints in vivo, we injected mAlb-CTLA4Ig (220 μg) into normal or CIA mice for 24 h. The mice were killed, and the paws were harvested and homogenized in RIPA buffer by mortar and pestle in liquid nitrogen. The degree of digestion was then analyzed by reducing SDS-PAGE and western blotting using an anti-mouse CTLA4 antibody.

### Binding activity of Alb-CTLA4Ig before and after MMP digestion

Ninety-six-well plates were precoated with polylysine (10 μg/ml) for 10 min. HEK-293 cells (10^5^ cells/well) stably expressing mouse or human CD80 (Additional file 2: Supplementary methods) were seeded into plates in DMEM supplemented with 10% fetal bovine serum overnight. The cells were washed in PBS and fixed with 4% paraformaldehyde in PBS at room temperature for 30 min. The cells were washed twice in PBS and blocked for nonspecific binding by 1% nonfat milk in PBST for 1 h. The indicated amounts of nondigested, MMP-digested Alb-CTLA4Ig or conventional CTLA4Ig were added to the cells for 1 h, followed by sequential addition of HRP-conjugated anti-mouse IgG (H+L) or anti-human Fcγ antibodies (Jackson ImmunoResearch Laboratories) and a chromogenic substrate TMB (Thermo Fisher Scientific). The optical density was measured at 450 nm in a microplate reader (Molecular Devices, San Jose, CA, USA). To estimate the concentrations that give rise to half-maximal binding (EC_50_), we plotted the absorbance at 450 nm against protein concentrations using a four-parameter logistic fit equipped with the GraphPad Prism software (GraphPad, San Diego, CA, USA).

### Blockade of Jurkat T cell activation by hAlb-CTLA4Ig before and after MMP digestion

Human Jurkat T cells (Bioresource Collection and Research Center, Hsin-Chu, Taiwan) were activated by anti-CD3 antibodies (BioLegend) and Raji cells (to provide CD80 costimulation [28]) (Bioresource Collection and Research Center). In brief, 10^5^ Jurkat T cells were premixed with 50 ng/ml anti-CD3 antibodies at 37 °C for 15 min and then cocultured with 10^5^ Raji B cells premixed with the indicated amounts of nondigested hAlb-CTLA4Ig, MMP-digested hAlb-CTLA4Ig, or conventional human CTLA4Ig (hCTLA4Ig) in 96-well plates for 24 h. Interleukin-2 (IL-2) secreted by Jurkat T cells was measured by a human IL-2 ELISA kit (BioLegend). To estimate the concentrations that inhibit half-maximal IL-2 secretion (IC_50_), we plotted IL-2 concentrations against concentrations of conventional hCTLA-4Ig, nondigested or MMP-digested hAlb-CTLA-4Ig, using a four-parameter logistic fit.

### Treatment efficacy of mAlb-CTLA4Ig for mouse collagen-induced arthritis

Collagen-induced arthritis (CIA) was induced as previously described [29]. Treatment of CIA was initiated when the disease score reached 1 in any paw of the mouse. PBS alone, mCTLA4Ig (1 nanomole), or mAlb-CTLA4Ig (1 nanomole) was intravenously injected into the mice (*N* = 6 per group) every 3 days for 4 injections. The CIA scores of treated mice were recorded every 3 days as described previously [30]. Mice were killed after therapy experiments, and the paws were collected, fixed, and decalcified in Decalcifier I solution (Leica Biosystems, Buffalo Grove, IL, USA) for histopathological examinations.

### Safety of mAlb-CTLA4Ig for treating mouse collagen-induced arthritis

Splenocytes from PBS-, mCTLA4Ig-, or mAlb-CTLA4Ig-treated mice were isolated after the therapy experiments. Splenocytes were seeded into 24-well plates (10^6^/well) and stimulated with 25 μg/ml *M. tuberculosis* extracts (Additional file 2: Supplementary methods) for 72 h. BrdU (5-bromo-2′-deoxyuridine) was added to the splenocytes at a concentration of 10 μM during the last 2 h of stimulation. The splenocytes were harvested and stained for cell surface markers (CD45, B220, CD3e, BioLegend) and then stained with a FITC-conjugated anti-BrdU antibody following the manufacturer’s manual (BD Bioscience). The proliferating splenocytes were then analyzed and quantified by a flow cytometer (C6, BD Bioscience).

### Statistical analyses

Concentrations of IL-2 and TNF-α and percent splenocyte proliferation are expressed as the mean ± SD. One-way ANOVA was used to detect differences in IL-2, TNF-α concentrations, or proliferating splenocyte subsets (CD45^+^, CD3e^+^, or B220^+^) among treatment groups, followed by Tukey’s tests for pairwise comparisons. CIA disease scores are expressed as the mean ± SEM. The nonparametric Friedman test was used to compare the treatment efficacies among groups, followed by the Conover test with Bonferroni correction for pairwise comparisons.

## Results

### Design and production of mAlb-CTLA4Ig

**Fig. 2:**
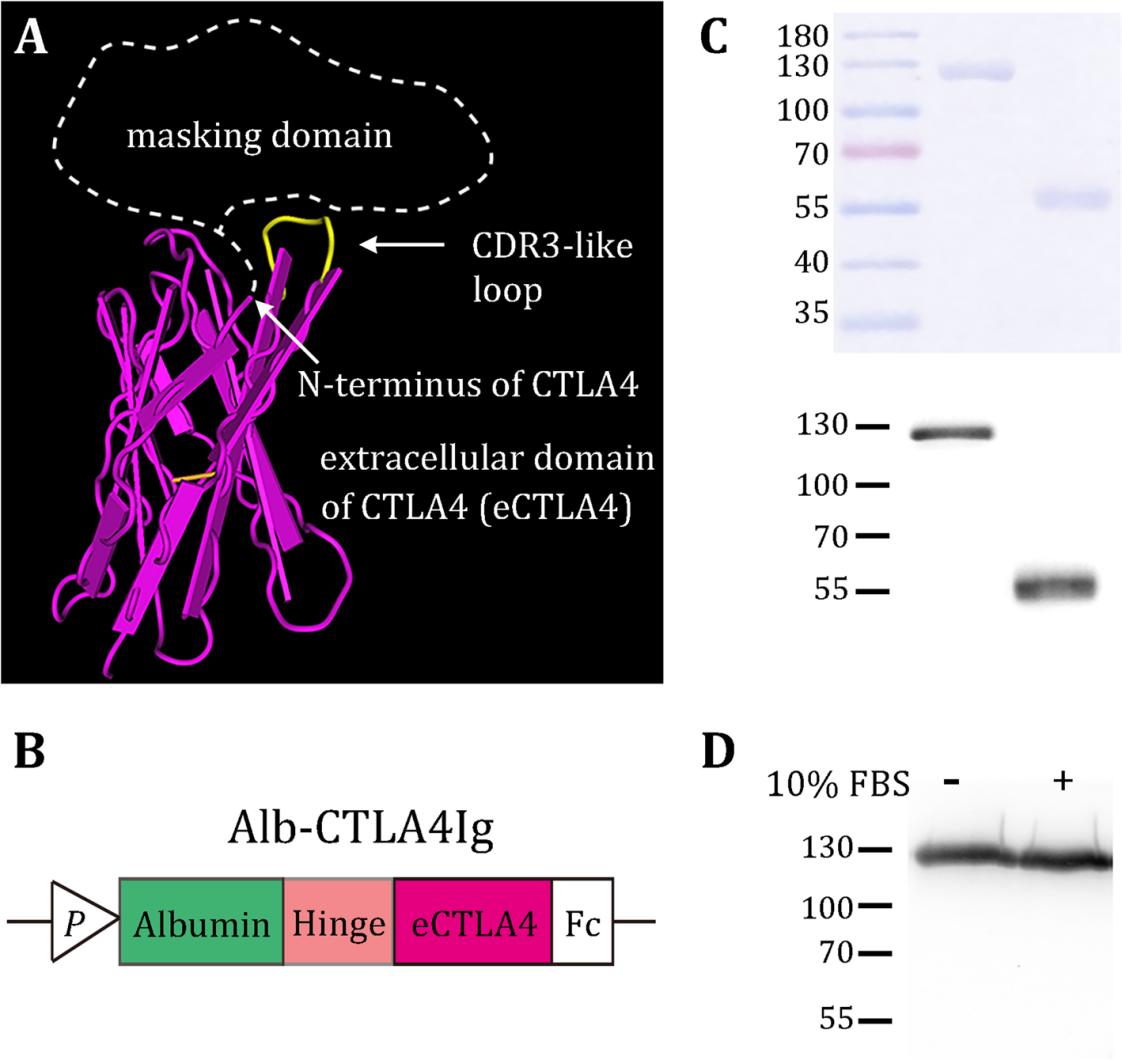
Design, production, and stability of mAlb-CTLA4Ig. **A** Structure of the extracellular domain of mCTLA4 (pink). The CDR3-like loop is marked in yellow. To block the binding activity, an N-terminal masking domain (dashed shape) should occupy the space over the CDR3-like loop. **B** Schematic representations of mAlb-CTLA4Ig constructs. eCTLA4, extracellular domain of CTLA4; P, promoter in the expression vector. **C** Reducing SDS-PAGE (upper panel) and western blot (lower panel, detected by an anti-mouse Fcγ antibody) analyses of mAlb-CTLA4Ig (left lane) and mCTLA4Ig (right lane) purified from ExpiCHO cells transfected with the expression vectors. **D** The stability of Alb-CTLA4Ig in DMEM containing 10% fetal bovine sera for 7 days, analyzed by western blot using an anti-mouse Fcγ antibody

We explored the crystal structures of mouse and human CTLA4 from the Protein Database (PDB ID: 1DQT [31] and 3OSK [32], respectively). The complementarity-determining region (CDR) 3-like loop (marked in yellow, Fig. 2A), a motif required for CD80/CD86 binding [31], is spatially adjacent to the N-terminus of the extracellular domain (ECD) of CTLA4. This spatial organization led us to speculate that if we link a masking domain to the N-terminus of CTLA4 (dashed line, Fig. 2A), we may block the accessibility of the CDR3-like loop to CD80/CD86. To this aim, we initially used the “Ab lock” [33] as a masking domain for mCTLA4Ig, given that CTLA4 ECD and antibody domains are structurally similar. However, the Ab lock (PRGPTIKPCPPCKCP of mouse IgG_2a_) at the N-terminus of mCTLA4Ig did not block its binding activity (Additional file 1: Fig. S1A). Next, we fused the mouse globulin iota chain (VpreB) to the N-terminus of mCTLA4Ig, considering that VpreB is a surrogate light chain capable of pairing to numerous newly rearranged heavy chains in B cell development [34]. Unfortunately, this attempt was not successful (Additional file 1: Fig. S1B).

We thus reasoned that a successful masking domain should cover a sufficient area over the CDR3-like loop. We finally turned our attention to albumin because it is considerably larger than the previous pro domains and is approved by the FDA for human use. We linked mouse albumin, via a core hinge-lower hinge/CH2 region sequence of IgG_2a_ (CPPCKCPAPNLLGGP) [35], to the N-terminus of mCTLA4Ig to construct mAlb-CTLA4Ig (Fig. 2B). We hypothesized that albumin has sufficient volume to block the accessibility of the CDR3-like loop to CD80/CD86, yet cleavage on the hinge sequence by MMP2/9 would remove albumin from mCTLA4Ig to restore its activity.

We purified mAlb-CTLA4Ig from the culture supernatants of ExpiCHO cells transiently transfected with the expression constructs. mAlb-CTLA4Ig is a dimeric protein that under reducing conditions it displayed a slightly larger-than-expected (111 kDa) molecular weight of ~ 130 kDa (left lane, Fig. 2C) due to glycosylation on Fc and CTLA4. For comparison, CTLA4Ig (also a dimeric protein) displayed a monomeric molecular weight of ~ 55 kDa under reducing conditions (right lane, Fig. 2C). Incubation of purified mAlb-CTLA4Ig in 10% fetal bovine sera for 7 days did not cause decomposition of the recombinant proteins, as no remarkable change in the molecular size was noted (Fig. 2D). These data indicate that mAlb-CTLA4Ig is stable in physiological conditions such as the sera.

### Digestion of mAlb-CTLA4Ig by MMP2/9 and restoration of its binding activity

**Fig. 3:**
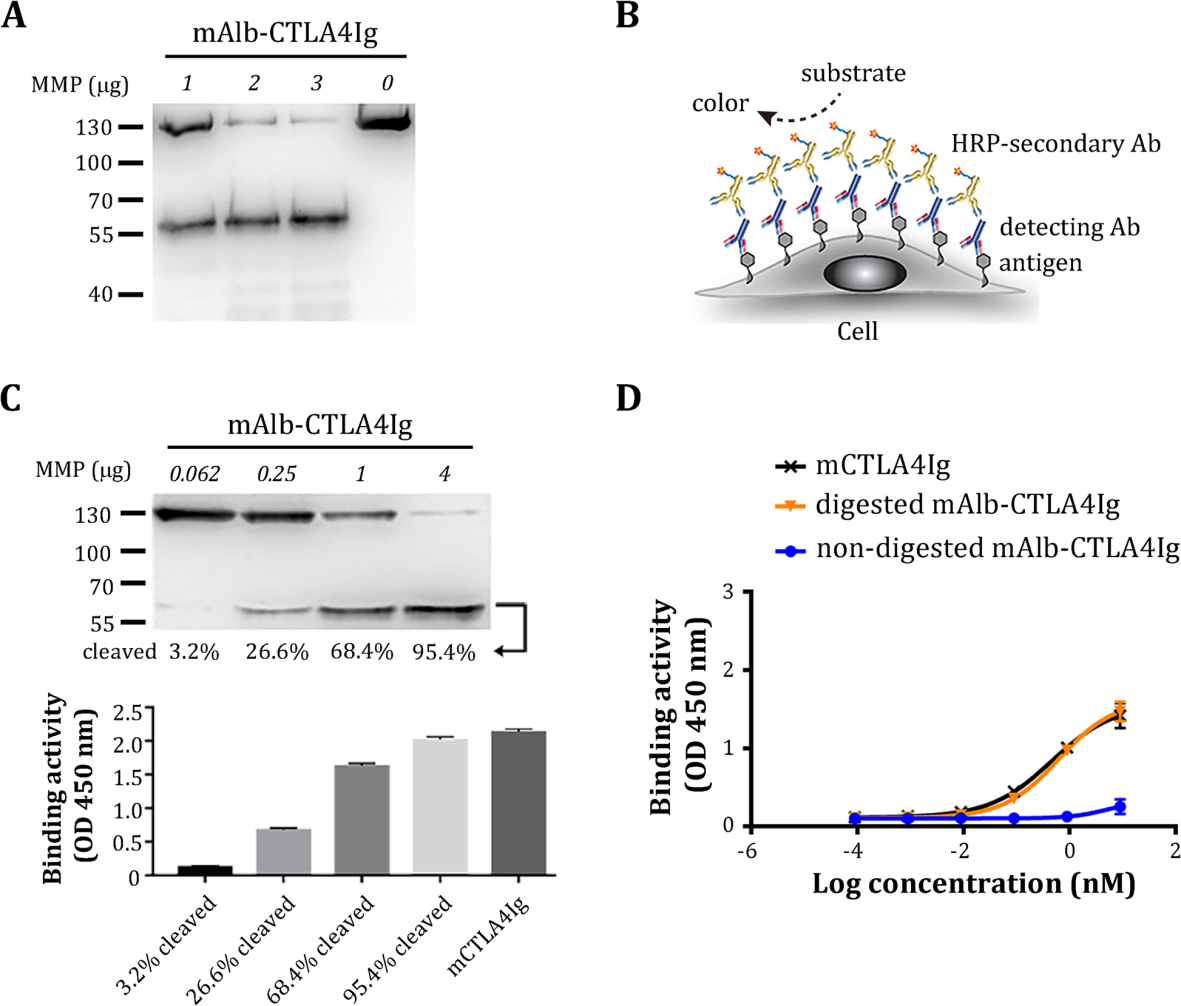
Binding activities of mAlb-CTLA4Ig before and after MMP2/9 digestion. **A** mAlb-CTLA4Ig was digested with the indicated amount of MMP2/9 and then analyzed by western blot using an anti-mouse Fcγ antibody. Note that the major bands after digestion correspond to the size of conventional mCTLA4Ig (~ 55 kDa under reducing conditions). **B** Schematic representation of the cell-based ELISA used in the current study. **C** mAlb-CTLA4Ig were subjected to varying degrees of digestion by MMP2/9. Part of the digestion was analyzed by western blot to determine the degree of cleavage. The percent (%) cleaved Alb-CTLA4Ig was quantitated by the ImageJ software and is indicated below each lane. The other part of the digestion was added to the cell-based ELISA. The binding of CD80 to 1 nM conventional mCTLA4Ig was used as a positive control. **D** mAlb-CTLA4Ig was digested by MMP2/9. Graded amounts (10-fold serial dilution) of nondigested (blue curves) or MMP-digested (orange curves) mAlb-CTLA4Ig were tested for their binding kinetics in the cell-based ELISA. The binding kinetics of conventional mCTLA4Ig are shown as a black curve

We tested whether mAlb-CTLA4Ig could be digested by MMP2/9 (type IV collagenase), which are highly upregulated in rheumatoid arthritis [36]. As expected, MMP2/9 cleaved mAlb-CTLA4Ig fusion proteins, reducing their molecular size from ~ 130 to ~55 kDa (Fig. 3A).

We next tested the binding activities of mAlb-CTLA4Ig before and after removing the N-terminal albumin by MMP2/9 using a cell-based ELISA, as illustrated in Fig. 3B. Nondigested mAlb-CTLA4Ig binds very weakly to mouse CD80 expressed on HEK-293 cells. However, binding activities were completely restored after mAlb-CTLA4Ig was digested with MMP2/9 (Additional file 1: Fig. S2). Furthermore, the binding activity in the ELISA increased proportionally with the extent of digestion (% cleaved) of mAlb-CTLA4Ig (Fig. 3C).

We further compared the binding kinetics of nondigested and digested mAlb-CTLA4Ig using cell-based ELISA. The binding curve of nondigested mAlb-CTLA4Ig is conspicuously right-shifted and remains very low even at high concentrations, indicating weak binding activity. We could not estimate the EC_50_ since the signal intensity of nondigested mAlb-CTLA4Ig did not reach the half-point of the signal plateau at the highest amount used in the assay (Fig. 3D). In contrast, the binding curve of MMP-digested mAlb-CTLA4Ig is comparable to that of conventional mCTLA4Ig (EC_50_ = 0.63 nM and 0.47 nM, respectively). In another construct, an MMP2/9 substrate sequence (GPLGVR) [16] was used to link albumin to mCTLA4Ig (we termed mAlb-MMP-CTLA4Ig in Additional file 1: Fig. S3). This construct displayed a slightly less difference in binding activity between nondigested and MMP-digested forms (Additional file 1: Fig. S3). Thus, it appeared to us that the hinge sequence is a superior linker to construct lesion-selective Alb-CTLA4Ig.

### An N-terminal albumin and a C-terminal Ig domain for optimal masking efficiency and stability

Since the placement of a sufficiently large protein domain, such as albumin, could successfully block the binding activity of CTLA4, we next tested whether placing the Ig domain, which is approximately 2-fold larger than VpreB, to the N-terminus of the extracellular domain (ECD) of CTLA4 (*i.e., m*Ig-CTLA4 ECD) could also block its binding activity. We found that this conformation moderately masked the binding activity of the mCTLA4 ECD (Additional file 1: Fig. S4A). However, mIg-CTLA4 ECD seemed more sensitive to protease digestion, as nearly complete degradation was revealed by western blot analysis (Additional file 1: Fig. S4A). This observation led us to determine whether removing the C-terminal Ig domain in mAlb-CTLA4Ig (*i.e.*, mAlb-CTLA4 ECD) could also lead to increased protease digestion of the mCTLA4 ECD. Indeed, heightened digestion was noted when mAlb-CTLA4 ECD was incubated with MMP2/9 (Additional file 1: Fig. S4B). Thus, based on these results, we conclude that N-terminal albumin is required for optimal masking efficiency and that the C-terminal Ig domain is required for its stability. Interestingly, conventional hCTLA4Ig also seemed susceptible to MMP cleavage, presumably at the hinge region connecting the hCTLA4 ECD and the Ig domain (Additional file 1: Fig. S4C). Whether the hCTLA4Ig used in the clinics is cleaved in vivo and whether digestion of hCTLA4Ig by MMPs in the inflammatory site would hamper its therapeutic efficacy are unknown. Overdigestion of hAlb-CTLA4Ig could occur if higher amounts of the enzymes are added (Additional file 1: Fig. S4D); however, the ~ 55-kDa band (converted hCTLA4Ig) seems to be the dominant cleavage product in the overdigestion.

### Binding activity and biological activity of hAlb-CTLA4Ig

**Fig. 4:**
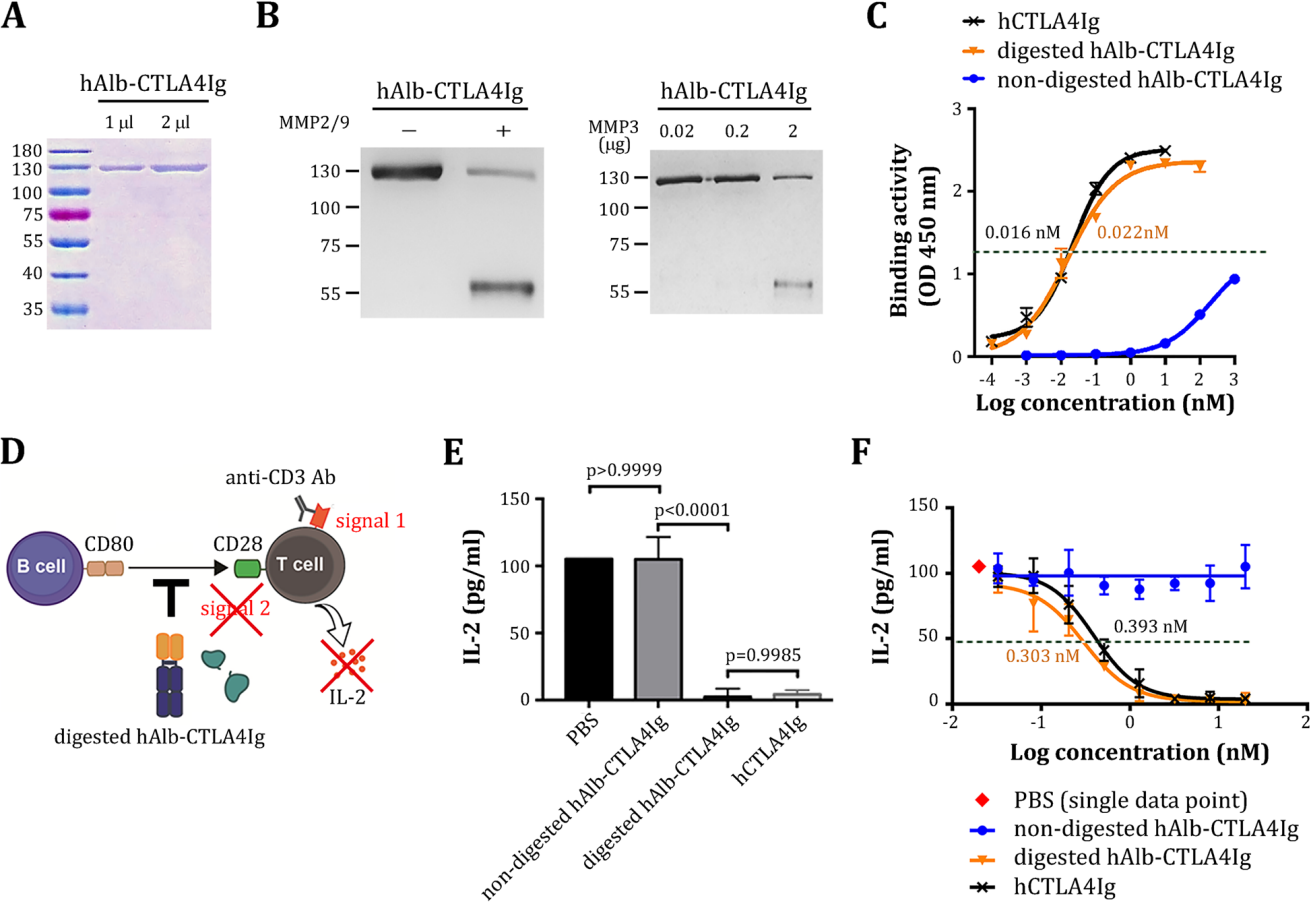
Production, binding kinetics, and biological activity of hAlb-CTLA4Ig. **A** Purity assessment of hAlb-CTLA4Ig by SDS-PAGE and Coomassie blue staining after purification of the recombinant proteins from the culture supernatant of ExpiCHO transfectants. **B** Cleavage of hAlb-CTLA4Ig by MMP2/9 (left panel) and MMP-3 (right panel) was assessed by western blot using a goat-anti human IgG antibody. **C** Binding kinetics of nondigested hAlb-CTLA4Ig (blue curve), MMP-digested (orange curve) hAlb-CTLA4Ig, or conventional hCTLA4Ig (black curve) were analyzed using cell-based ELISA. EC_50_ values are indicated with matching colors. **D** Schematic representation of the in vitro human T cell activation model in the current study. **E** Effects of nondigested hAlb-CTLA4Ig, MMP-digested hAlb-CTLA4Ig, or conventional hCTLA4Ig (all at 1 nM) on IL-2 secretion by Jurkat T cells. **F** Inhibition curves of Jurkat T cell activation by nondigested hAlb-CTLA4Ig (blue curve), MMP-digested hAlb-CTLA4Ig (orange curve), or conventional hCTLA4Ig (black curve). IC_50_ values are indicated by matching colors

We extended the design concept to construct and test hAlb-CTLA4Ig using a core hinge-lower hinge/CH2 region sequence (CPPCPAPELLGGP) [37] of a human immunoglobulin to link albumin and hCTLA4Ig. Using protocols similar to those described above, we produced and purified hAlb-CTLA4Ig to near homogeneity (Fig. 4A). hAlb-CTLA4Ig was stable in a medium containing 10% serum (Additional file 1: Fig. S5). We confirmed that hAlb-CTLA4Ig could be digested by MMP2/9, as indicated by a reduction in the protein size in the western blot analysis (Fig. 4B). In addition, hAlb-CTLA4Ig was also cleaved by MMP3, an inflammatory enzyme highly expressed in rheumatoid arthritis [38, 39]. Binding kinetics studies indicated that nondigested hAlb-CTLA4Ig had virtually no binding to human CD80 until at very high concentrations. However, its binding activity was restored by MMP2/9 digestion to a level similar to that of conventional hCTLA4Ig (EC_50_ = 0.016 nM and 0.022 nM for conventional hCTLA4Ig and MMP-digested hAlb-CTLA4Ig, respectively, Fig. 4C). We could not determine the actual EC_50_ for nondigested hAlb-CTLA4Ig but roughly estimated that it was at least 10,000-fold larger than the MMP-digested form.

We referred to a prior publication in which human Jurkat T cells were activated by anti-CD3 antibodies (signal 1) and costimulated by CD80 (signal 2) expressed on human Raji B cells [40]. In this in vitro model, we added nondigested or MMP-digested hAlb-CTLA4Ig to test whether they inhibit Jurkat T cell activation by blocking signal 2, thus reducing the production of interleukin-2 (IL-2) (Fig. 4D). We found that nondigested hAlb-CTLA4Ig (1 nM) did not inhibit IL-2 production by Jurkat T cells (Fig. 4E). However, after digestion with MMP2/9, its ability to inhibit IL-2 production was comparable to that of conventional hCTLA4Ig (both at 1 nM, Fig. 4E). Finally, we determined and compared the IC_50_ of nondigested and MMP-digested hAlb-CTLA4Ig in inhibiting the activation of Jurkat T cells. Nondigested hAlb-CTLA4Ig did not inhibit IL-2 production at any dose tested (Fig. 4F), indicating that its biological activity is masked by albumin. In contrast, MMP-digested hAlb-CTLA4Ig displayed a similar inhibition curve as conventional hCTLA4Ig (IC_50_ = 0.303 nM and 0.393 nM for MMP-digested hAlb-CTLA4Ig and conventional hCTLA4Ig, respectively, Fig. 4F), indicating a complete restoration of biological activity of hAlb-CTLA4Ig after MMP digestion.

### Structural simulation of mAlb-CTLA4Ig

**Fig. 5:**
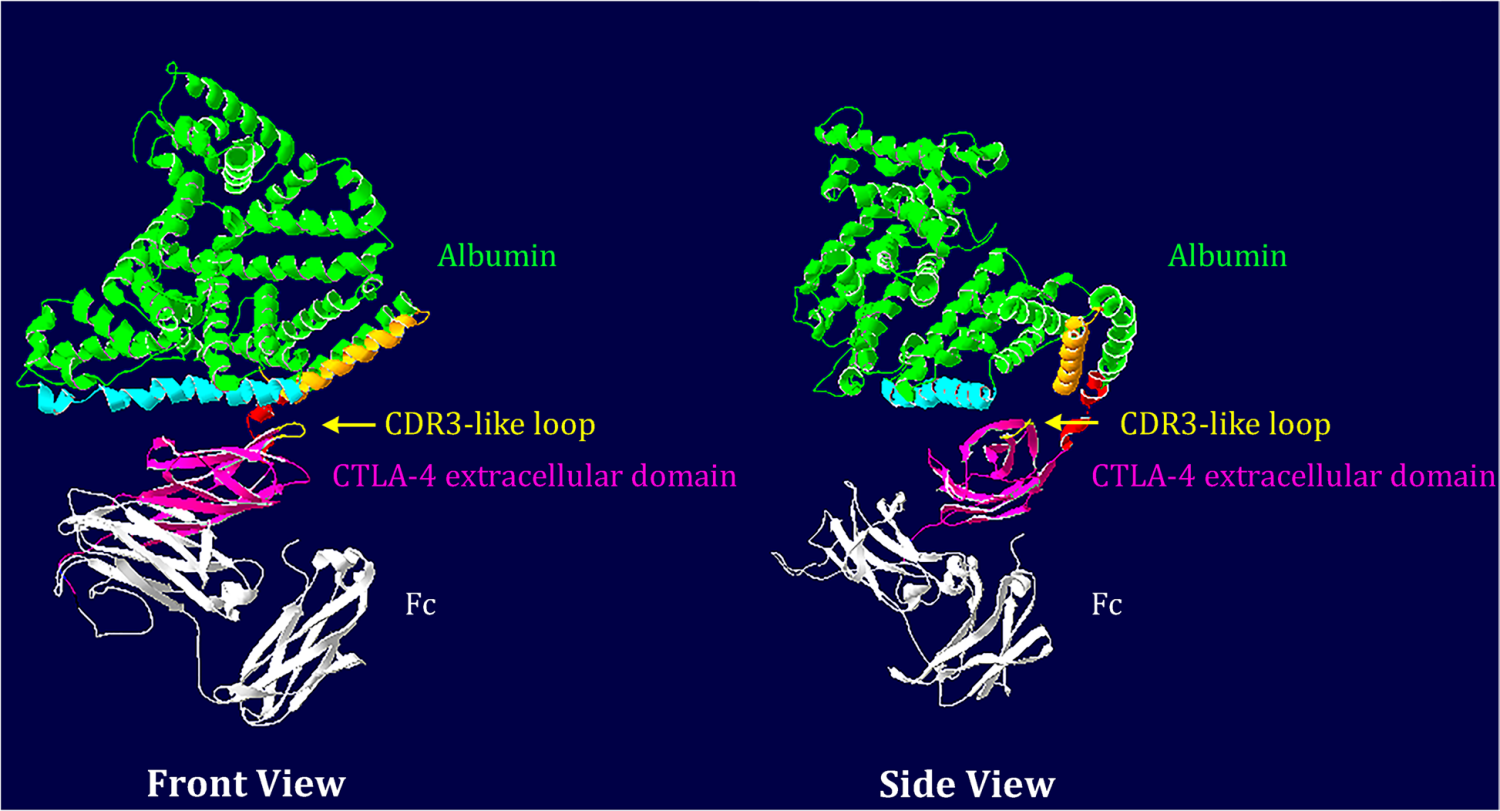
Structure simulation of mouse Alb-CTLA4Ig. The protein sequences of mAlb-CTLA4Ig were input into the online server ROBETTA for structure prediction. Shown here are the front view and the side view (rotated 90°) of the predicted structure of the fusion protein where albumin is represented in green, the extracellular domain of mouse CTLA4 is represented in magenta, the mouse Ig domain is represented in white, and the MMP substrate sequence is represented in red. Note that the CDR3-like loop is blocked by helix h4 (light blue) of domain IIb and helix h3 (orange) of domain IIIb

We used structural simulations to explain how albumin may have masked the binding activity of mCTLA4Ig using the online structure prediction server ROBETTA [41]. A predicted structure of mAlb-CTLA4Ig is shown in Fig. 5. Based on the predicted structure, albumin seems to occupy a large space over the top of the CDR3-like loop. It is noted that the CDR3-like loop is blocked by helix h4 (Pro367-Leu398) of domain IIb and helix h3 (Lys538-Asp563) of domain IIIb of albumin [42]. This structural modeling helps us to explain the potential mechanism of action imposed by albumin, although the actual structure of mAlb-CTLA4Ig remains to be solved experimentally.

### Activation of mAlb-CTLA4Ig in the joints of mice that developed collagen-induced arthritis

**Fig. 6:**
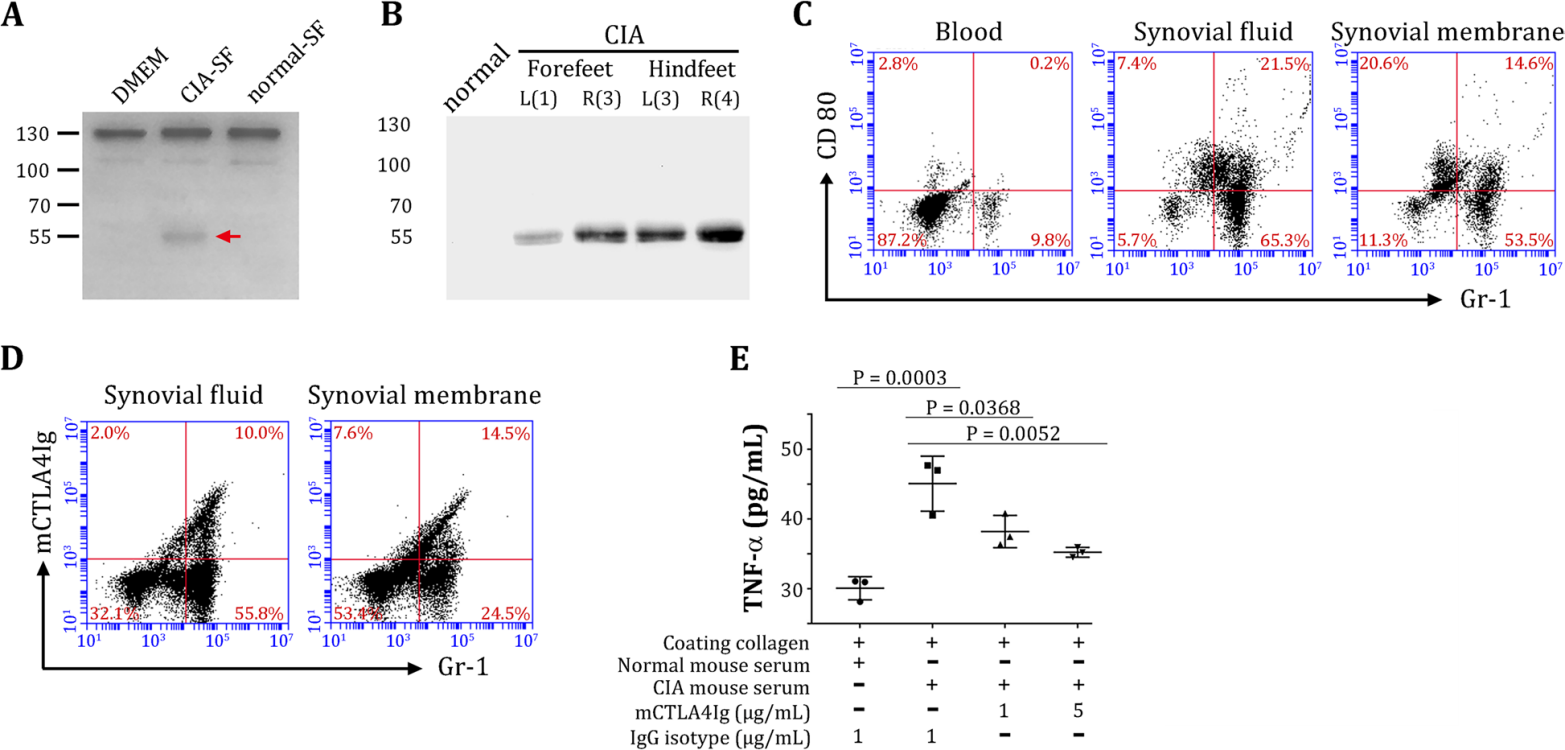
Conversion of mAlb-CTLA4Ig to mCTLA4Ig in the joints of mice that developed collagen-induced arthritis (CIA). **A** mAlb-CTLA4Ig was added to DMEM, CIA synovial fluid (CIA-SF) lavage, or normal synovial fluid (normal-SF) lavage. Conversion of mAlb-CTLA4Ig (~ 130 kDa under reducing condition) to mCTLA4Ig (red arrow, ~ 55 kDa under reducing condition) was detected by western blot using an anti-mouse CTLA4 antibody. **B** mAlb-CTLA4Ig was injected into CIA mice or normal mice for 24 h. Paws were harvested, homogenized, and analyzed by western blotting using an anti-mouse CTLA4 antibody. Positions of paws are indicated by capitalized letters R and L. Numbers in parentheses indicate the disease scores of the paws. **C** Synovium-infiltrating cells were isolated from CIA mice and analyzed for Gr-1 and CD80 expression by flow cytometry. Approximately 1/3 of the GR-1^+^ cells in the synovial fluid and ~ 1/5 of the GR-1^+^ cells in the synovial membranes expressed CD80, whereas blood GR-1^+^ cells did not express CD80. **D** Synovium-infiltrating cells were isolated from CIA mice and tested for mCTLA4Ig binding by flow cytometry. **E** Effect of in vitro mCTLA4Ig treatments on TNF-α secretion by joint-infiltrating cells stimulated with precoated collagen-antibody immunocomplexes

We tested whether synovial fluids aspirated from the joints of mice that developed collagen-induced arthritis (CIA) would contain enough MMPs to digest mAlb-CTLA4Ig to mCTLA4Ig. Incubation of mAlb-CTLA4Ig with CIA synovial lavage (CIA-SF) resulted in a mild conversion of mAlb-CTLA4Ig to an ~ 55-kDa band (approximately 8%), which is the expected molecular weight of mCTLA4Ig (Fig. 6A) under reducing electrophoresis. It should be emphasized that synovial lavage represents a “diluted” form of synovial fluid since we aspirated the fluids after injecting DMEM into the joints; the actual CIA synovial fluids would have contained higher MMP activities per unit volume. In contrast, mAlb-CTLA4Ig incubated in normal synovial lavages (normal-SF) remained intact (~ 130 kDa).

To test whether mAlb-CTLA4Ig could be converted to mCTLA4Ig in inflammatory tissues in vivo, we intravenously injected mAlb-CTLA4Ig into normal mice or mice that developed CIA. We found converted mCTLA4Ig (~ 55 kDa) in the paws of CIA mice 24 h after injection of mAlb-CTLA4Ig but not in the paws of normal mice (Fig. 6B). Interestingly, the signal intensity seems to correlate with the disease scores of the paws (disease scores are indicated in the parentheses for each paw in Fig. 6B), suggesting that higher levels of MMPs might be expressed in the more inflamed joints to digest and convert mAlb-CTLA4Ig to mCTLA4Ig. Thus, these data indicate that nonactive mAlb-CTLA4Ig could be converted to therapeutic mCTLA4Ig in inflammatory but not normal joints. We did not observe nondigested Alb-CTLA4Ig in the protein extracts of paws of both normal control mice and CIA mice, possibly due to low Alb-CTLA4Ig concentration in the interstitial fluids/blood at 24 h post-injection, and that Alb-CTLA4Ig become further diluted due to abundant proteins released from tissues during the extraction process. In contrast, when incoming Alb-CTLA4Ig is digested, its abundance could accumulate in the synovia by binding to CD80 on the infiltrating cells.

To validate that inflamed joints express high levels of MMPs, we measured MMP9 and MMP3 proteins in the synovial fluid lavages, sera, and paws of CIA mice by ELISA (Additional file 2: Supplementary methods). We found that MMP9 concentrations in the synovial fluid lavages (682.4 ng/ml vs. 18.6 ng/ml) and paws (3805.9 ng/mg protein vs. 139.2 ng/mg protein) were markedly higher in CIA mice than that in normal control mice (Additional file 1: Fig. S6A). Although MMP3 expression levels were lower in both CIA mice and normal control mice, a similar trend of MMP3 concentrations was noted in the synovial fluid lavages (6.3 ng/ml vs. 0.9 ng/ml) and paws (44.4 ng/mg protein vs. 2.1 ng/mg protein) (Additional file 1: Fig. S6B). In contrast, MMP9 (27.2 ng/ml vs. 19 ng/ml) and MMP3 (2.3 ng/ml vs. 12.6 ng/ml) in the sera were low and comparable between CIA mice and normal control mice. Collectively, these data support the notion that the digestion of Alb-CTLA4Ig into active CTLA4Ig occurred in situ in the inflamed joint but not in the sera.

To gain more insight into the targeting and activation of mAlb-CTLA4Ig in the inflamed joints, we analyzed the infiltrating cells from the synovial fluids and synovial membranes by flow cytometry (Additional file 2: Supplementary methods). We noted that many joint-infiltrating cells in the synovial fluids and synovial membranes were neutrophils (Gr-1^+^), consistent with previous findings [43]. Approximately 1/3 of the joint-infiltrating neutrophils, but not blood neutrophils, expressed CD80 (Fig. 6C). In addition, these cells could bind to conventional mCTLA4Ig (Fig. 6D). Interestingly, in vitro treatments (Additional file 2: Supplementary methods) of joint-infiltrating neutrophils with conventional mCTLA4Ig dampened the secretion of tumor necrosis factor-α (TNF-α) (Fig. 6E), a well-known pathogenic cytokine in human and mouse arthritis [1, 44, 45]. Collectively, these data indicate that mAlb-CTLA4Ig can be targeted and activated in inflamed joints to treat inflammation.

### Efficacy and safety of mAlb-CTLA4Ig in treating collagen-induced arthritis

**Fig. 7:**
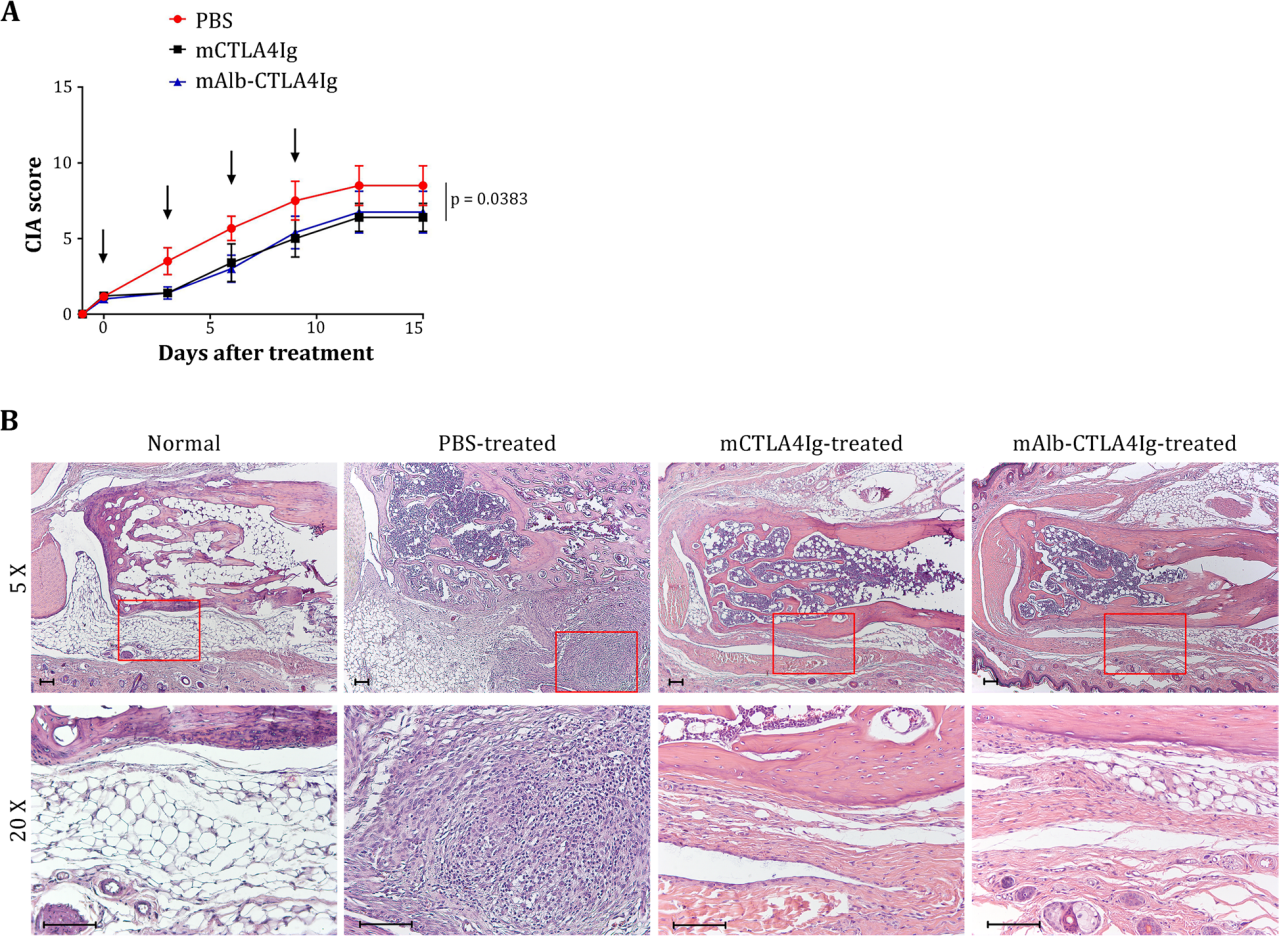
Efficacy of mAlb-CTLA4Ig for treating mouse collagen-induced arthritis. **A** Treatment efficacies of mAlb-CTLA4Ig and mCTLA4Ig on CIA. Arrows indicate the time of treatments. Representative data from two independent experiments are shown. **B** Histopathological microphotographs of the paws of a normal mouse or CIA mice that received different treatments. Areas enclosed by red rectangles (× 5 objective, upper row) are shown at higher magnification (× 20 objective, lower row) for inflammatory cells. Scale bar: 100 µm

It was previously shown that CTLA4Ig could ameliorate the severity of mouse CIA when the treatment was initiated after the mice developed clinical signs [46–49]. As shown in Fig. 7A, the disease score (CIA score) steadily increased in CIA mice treated with PBS. However, disease was suppressed when CIA mice were treated with mCTLA4Ig or mAlb-CTLA4Ig (*p* = 0.0383, Friedman test for repeated measures, PBS vs. mCTLA4Ig, *p* = 0.013618; PBS vs. mAlb-CTLA4Ig, *p* = 0.013618; mCTLA4Ig vs*.* mAlb-CTLA4Ig, *p* > 0.9999). Histologically, numerous inflammatory cells (mostly neutrophils and reactive fibroblast-like cells) were found in the ankles and digits of PBS-treated CIA mice. These inflammatory cells infiltrated the synovial membranes and connective tissues underneath the skin in the paws of PBS-treated CIA mice. In addition, inflammatory cells aggregated into a nodule-like structure (Fig. 7B). In contrast, the infiltration of inflammatory cells was largely reduced in the ankles of mAlb-CTLA4Ig- and mCTLA4Ig-treated CIA mice (Fig. 7B), while inflammation in the digits was similar in all groups (Additional file 1: Fig. S7). These data indicate that mAlb-CTLA4Ig is as efficient as conventional mCTLA4Ig in treating mouse CIA, although neither cannot cure the disease.

**Fig. 8:**
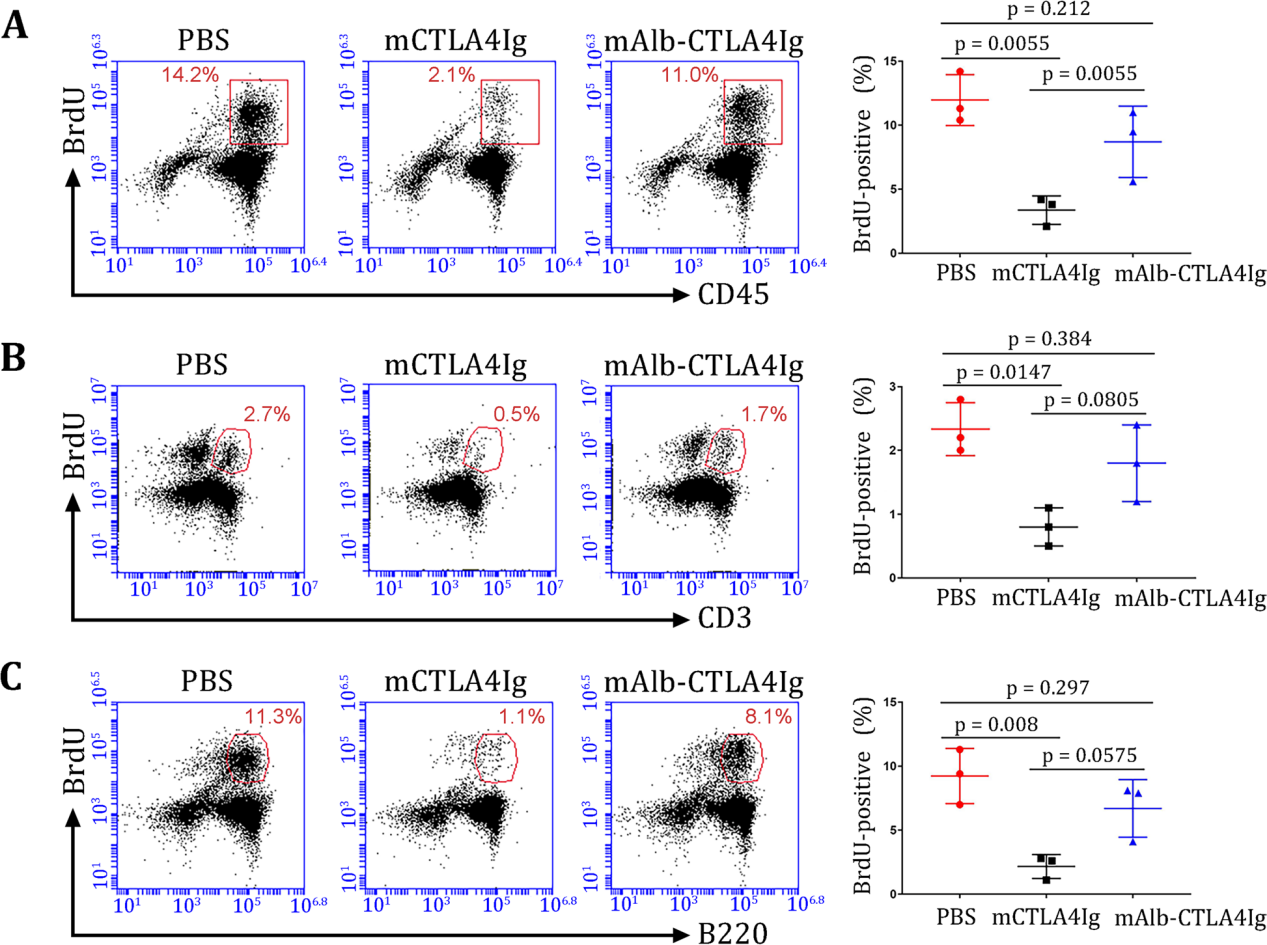
Safety of Alb-CTLA4Ig. Splenocytes from PBS-, mCTLA4Ig-, or mAlb-CTLA4Ig-treated CIA mice were stimulated with 25 μg/ml *M. tuberculosis* extracts for 72 h. Proliferation (BrdU incorporation) of CD45^+^ splenocytes (**A**), CD3^+^ T cells (**B**), and B220^+^ B cells (**C**) was analyzed by flow cytometry. Representative data from two independent experiments are shown

Complete Freund’s adjuvants used for the induction of CIA contain heat-inactivated *M. tuberculosis*. Therefore, the immune responses against *M. tuberculosis* were activated in these mice, as shown by the proliferation of splenocytes (Additional file 1: Fig. S8). Since mAlb-CTLA4Ig is designed to reduce systemic immune suppression, we investigated whether mAlb-CTLA4Ig selectively suppresses joint inflammation but not the immune responses against *M. tuberculosis* in the spleen. A substantial proportion of splenocytes isolated from PBS-treated CIA mice proliferated in response to 25 μg/ml *M. tuberculosis* extracts (Fig. 8A). Splenocyte proliferation was reduced by 3.65-fold (PBS 11.33% vs. mCTLA4Ig 3.1%, *p* = 0.0055) when CIA mice were treated with conventional mCTLA4Ig. However, splenocytes from mAlb-CTLA4Ig-treated mice proliferated to a degree comparable to that observed in PBS-treated CIA mice (PBS vs. mAlb-CTLA4Ig, *p* = 0.212; mAlb-CTLA4Ig vs. mCTLA4Ig, *p* = 0.0055, Fig. 8A). Further examination of the proliferating subsets of lymphocytes in the spleen indicated that the proliferation of T cells was suppressed in mCTLA4Ig-treated mice but largely recovered in mAlb-CTLA4Ig-treated mice (PBS vs. mAlb-CTLA4Ig, *p* = 0.384; PBS vs. mCTLA4Ig, *p* = 0.0147; mAlb-CTLA4Ig vs. mCTLA4Ig, *p* = 0.0805, Fig. 8B). Similar results were found for the proliferation of B cells in these mice (PBS vs. mAlb-CTLA4Ig, *p* = 0.297; PBS vs. mCTLA4Ig, *p* = 0.008; mAlb-CTLA4Ig vs. mCTLA4Ig, *p* = 0.0575, Fig. 8C). Thus, mAlb-CTLA4Ig did not inhibit immune responses against *M. tuberculosis* in these mice, indicating that mAlb-CTLA4Ig might be a safe therapy for CIA.

## Discussion

Autoimmune diseases such as rheumatoid arthritis have no cure thus far but require long-term medical management. The use of biological disease-modifying antirheumatic drugs are still associated with adverse various effects [50]. Thus, the safety of long-term use of immunosuppressive drugs is an important concern in treating patients. To address this issue, we took advantage of the MMPs overexpressed in inflammatory tissues as a molecular switch to turn on the action of Alb-CTLA4Ig. Lesion-selective activation of therapeutic agents may reduce adverse effects and prevent premature binding of the therapeutic agents to ligands expressed in nondiseased tissues to improve the outcome of the therapy. We show that Alb-CTLA4Ig was converted to CTLA4Ig to suppress inflammation in the joints but not the antimicrobial immune responses in the spleens. This strategy may be extended for other inflammatory diseases where lesion-site selective action of CTLA4Ig is desirable. Therefore, Alb-CTLA4Ig may serve as a safer yet equally effective therapy for collagen-induced arthritis. Additional development and tests are required before human use.

Protein engineering methods to mask nonantibody therapeutic proteins require case-by-case trials. In contrast, generalized strategies have been developed for engineering lesion-selective antibodies [33]. For example, Ab lock using autologous hinge sequences [33, 51] or probody using bacterial display-identified peptides [52, 53] has been shown to mask the selected antibodies. While these methods are proven useful for antibodies, they may be inapplicable for nonantibody therapeutic proteins due to variations in structures and/or binding mechanisms. Therefore, effective methods to construct lesion-selective pro-proteins remain to be explored. Progress has been made in shielding cytokine actions to activate antitumor immunity. For example, the cytokine-binding homology region of IL-12 receptor β1 (the masking domain) was linked to the N-terminus of a hetero-IL-12-Fc via a cleavable MMP14 substrate sequence to form pro-IL-12. Nondigested pro-IL-12 showed 10-fold less activity than MMP14-digested pro-IL-12 in vitro, yet injection of pro-IL-12 achieved tumor suppression and reduced adverse effects in mice [54]. We used albumin to mask CTLA4Ig with great efficacy in the current study. Albumin is an endogenous serum protein; thus, linking albumin to CTLA4Ig should be safe and nonimmunogenic for human use. Considering that many members of the immunoglobulin superfamily (IgSF) are promising drug candidates, albumin may efficiently mask the binding sites of these IgSF members. Thus, our method could supplement the current methodology for developing lesion-selective therapeutic proteins.

We initially tried the Ab lock strategy to develop the functionally latent pro-CTLA4Ig, given that both CTLA4 and antibodies are members of the immunoglobulin superfamily with similar structures. This attempt failed, and molecular dynamics simulation indicated that the N-terminal Ab lock forms disulfide bonds in a location away from the CDR3-like loop (Additional file 1: Fig. S1C). Both the light chain and heavy chain contain 3 CDRs, and thus, the Ab lock may block any of the CDRs to mask the binding activity of the antibody. Unlike antibodies, the CDR3-like loop constitutes the only binding site for CTLA4, and the CDR3-like loop must be masked to block the binding activity. Our structure simulation using the ROBETTA algorithm supports this notion in that albumin covers a majority of the area, including the CDR3-like loop in CTLA4. This rationale suggests that albumin may efficiently mask the CDRs in antibodies, although we did not formally test this hypothesis.

Evidence indicates that extranodal (or secondary) activation of T cells occurs in chronically inflamed tissues such as rheumatoid arthritis and Hashimoto thyroiditis [55, 56]. We have also demonstrated that autoreactive T cells are reactivated in situ in the inflamed pituitary in human and mouse autoimmune hypophysitis [26]. In line with this notion, inflammatory antigen-presenting cells capable of stimulating synovial T cells were identified in patients’ joints in rheumatoid arthritis [57, 58], whereas CTLA4Ig was shown to block in vitro activation of synovial T cells by dendritic cells [59]. However, whether such secondary activation is required for the pathogenesis of autoimmune diseases is thus far unclear. In this regard, the lesion-selective Alb-CTLA4Ig may be a useful research tool to address the relative contribution of primary activation versus extranodal (secondary) activation of pathogenic T cells in the progression of autoimmune diseases. If confirmed, targeting secondary T cell activation may represent an attractive strategy to treat autoimmune diseases without the adverse effect caused by systemic T cell inhibition.

How CTLA4Ig may treat ongoing inflammation, such as rheumatoid arthritis, is debated. The current paradigm indicates that naïve T cells are activated in secondary lymphoid organs, and once activated, they exit secondary lymphoid organs to enter target tissues where further activation by resident APCs is no longer required before performing their functions. CTLA4Ig is thought to inhibit T cell activation in the secondary lymphoid organs, and as such, it is unlikely to inhibit pathogenic T cells in inflammatory tissues. However, as discussed above, some evidence indicates that the reactivation of T cells in chronically inflamed tissues may be critical in the pathogenesis of inflammatory diseases. On the other hand, CTLA4Ig could decrease disease severity in the absence of T cells in mouse CIA [47]. In such a scenario, CTLA4Ig may have direct effects on antigen-presenting cells. For example, CTLA4Ig can induce dendritic cells to express indoleamine 2,3-dioxygenase (IDO) [60], an enzyme known to downregulate T cell functions by degrading and depleting the tryptophan supply [61]. Our data suggest that CTLA4Ig may act locally in the inflammatory joints since many joint-infiltrating cells express CD80 to bind CTLA4Ig, and in vitro CTLA4Ig treatment reduced TNF-α secretion. In line with our results, it was previously shown that CD80, but not CD86, might be the primary target of CTLA4Ig [62]. Collectively, Alb-CTLA4Ig may be selectively activated in inflammatory tissues to inhibit pathogenic T cells by competing with CD80. Alternatively, activated Alb-CTLA4Ig may act on inflammatory cells by suppressing TNF-α production or inducing immune-modulatory molecules (such as IDO).

The current study focused on the development of Alb-CTLA4Ig and its efficacy and safety in treating CIA. We did not address whether Alb-CTLA4Ig could modulate autoantibodies (e.g., anti-citrullinated peptide antibody) production in the treated mice. Whether Alb-CTLA4Ig is effective in antibody-mediated arthritis models such as GPI-induced arthritis [63] may require further evaluation. It was shown that antigen-presenting cells (APCs) in the synovia migrate to the joint-draining lymph nodes [64]. The inflammatory milieu may promote this APC migration to draining lymph nodes where they present autoantigens to activate new T cells that later infiltrate the synovia or provide helps to B cells to produce new pathogenic antibodies. On the other hand, it was shown that CTLA4Ig reduced the surface expression of adhesion molecules and in vitro migration capacity of CD14+ monocytic cells [65]. Whether a lesion-selective action of CTLA4Ig in the inflamed synovia (such as in the case of Alb-CTLA4Ig) could inhibit APC trafficking to draining lymph nodes and subsequent antigen presentation to T cells may be interesting questions in the future.

## Conclusions

Our results indicate that N-terminal albumin very efficiently masked the activities of Alb-CTLA4Ig. Alb-CTLA4Ig may be MMP-converted to CTLA4Ig to suppress tissue inflammation in situ without hampering systemic antimicrobial responses. Lesion-selective activation of Alb-CTLA4Ig is expected to reduce adverse effects and antigen sinks. It is tempting to speculate that albumin may be a useful masking domain for constructing other pro-proteins in the immunoglobulin superfamily. Thus, our study may supplement the current engineering methods in constructing lesion-selective pro-proteins. Finally, Alb-CTLA-4Ig may be a useful research tool to address the mechanisms of CTLA4Ig treatment and whether extranodal T cell activation contributes to the pathogeneses of autoimmune diseases.

## Supplementary Information

**Additional file 1: Figure S1.** The N-terminal Ab lock and VpreB were unable to mask the binding activity of CTLA4Ig. **(A)** Binding activity of Ab lock-CTLA4Ig (0.5 μg/ml, blue line) and conventional CTLA4Ig (0.5 μg/ml, black line) to HEK-293 cells overexpressing CD80 (CD80 cells), detected by FITC-conjugated goat anti-mouse Fcγ in flow cytometry. Gray line: unstained cells. **(B)** Binding activity of VpreB-CTLA4Ig (0.5 μg/ml, blue line) and conventional CTLA4Ig (0.5 μg/ml, black line) to CD80 cells, detected by FITC-conjugated goat anti-mouse Fcγ antibodies by flow cytometry. Gray line: unstained cells. VpreB: immunoglobulin iota chain. **(C)** Simulation of Ab lock-mCTLA4Ig by the computer software BIOVIA Discovery Studio 2019 (Discovery Studio v19.1.0.18287). The structures of CTLA-4, the CDR3-like domain and the Ab lock are shown in magenta, yellow and light blue, respectively. **Figure S2.** Full recovery of the binding activity of mAlb-CTLA4Ig after MMP2/9 digestion. Nondigested, MMP-digested mAlb-CTLA44Ig, and conventional mCTLA4Ig (all at 1 nM) were added to the ELISA. Binding of the fusion proteins on the plate was detected by an HRP-conjugated anti-mouse IgG Fcγ secondary antibody. **Figure S3.** Characterization of an alternative Alb-CTLA4Ig with MMP substrate linker between albumin and CTLA4Ig (mAlb-MMP-CTLA4Ig). **(A)** Schematic representations of mAlb-MMP-CTLA4Ig constructs. MMP: MMP substrate sequence (GPLGMWSR) linker, eCTLA4: extracellular domain of CTLA4. P: promoter in the expression vector. **(B)** Reducing SDS-PAGE (left) and western blot analysis (right) of purified mAlb-MMP-CTLA4Ig. **(C)** The stability of mAlb-MMP-CTLA4Ig in DMEM containing 10% fetal bovine sera for seven days. **(D)** mAlb-MMP-CTLA4Ig were digested with the indicated amount of MMP2/9 and analyzed by western blot. **(E)** mAlb-MMP-CTLA4Ig were subjected to varying degrees of digestion by MMP2/9. Part of the digestion was analyzed by western blot to determine the degree of cleavage. The percent (%) cleaved Alb-MMP-CTLA4Ig was quantitated and is indicated below each lane. The digestion was added to the cell-based ELISA. The binding of CD80 by 1 nM conventional mCTLA4Ig was used as a positive control. **(F)** Binding kinetics of mAlb-MMP-CTLA4Ig before (blue curve) and after MMP digestion (orange curve). The binding kinetics of conventional mCTLA4Ig are shown in the black curve. **Figure S4.** Lacking an N-terminal albumin and a C-terminal Fc decreases masking efficiency and stability. **(A)** Competitive binding of Ig-CTLA4 ECD (0.5 μg/ml, blue line) or conventional CTLA4Ig (0.5 μg/ml, black line) against PE-conjugated anti-mouse CD80 antibodies (1 μg/ml) to HEK-293 cells overexpressing CD80 (CD80 cells). Red line: CD80-expressing cells stained with PE-conjugated anti-CD 80 antibodies alone (1 μg/ml). Gray line: unstained cells. Digestion products of the IgG1 Fc-CTLA4 ECD by the indicated amounts of MMP2/9 are shown in the western blot using anti-mouse Fcγ antibodies (right panel). **(B)** Competitive binding activity of Alb-CTLA4 ECD (0.5 μg/ml, blue line) and conventional CTLA4Ig (0.5 μg/ml, black line) to CD80 cells in the presence of PE-conjugated anti-mouse CD80 antibodies (1 μg/ml) by flow cytometry. Red line: CD80 cells stained with PE-conjugated anti-CD80 antibody alone (1 μg/ml). Gray line: unstained cells. Digestion products of the Alb-CTLA4 ECD by the indicated amounts of MMP2/9 are shown in the western blot using anti-mouse CTLA4 antibodies (right panel). **(C)** Digestion products of conventional human CTLA4Ig (3.6 picomole) by 2 units of MMP2/9 is shown in the western blot using anti-human Fcγ antibodies. **(D)** Overdigestion of hAlb-CTLA4Ig (1.35 picomole) in higher amounts (9 units) of MMP2/9. **Figure S5.** The stability of hAlb-CTLA4Ig in sera. hAlb-CTLA4Ig incubated in RPMI 1640 containing 10% fetal bovine sera for seven days was analyzed by western blot using an anti-human Fcγ secondary antibody. Med: medium alone. **Figure S6.** Differential levels of MMP9 and MMP3 in normal control mice and CIA mice. Protein levels of MMP9 **(A)** and MMP3 **(B)** in the synovial fluid lavages, sera, and paws of normal control mice and CIA mice were measured by ELISA. Protein levels are expressed as ng/ml in synovial fluid lavages and sera, ng/mg protein in protein extracts of the paws. **Figure S7.** Histopathological microphotographs of the digits of a normal mouse or CIA mice. The area enclosed by red rectangles at low magnification (5X objective, upper row) is shown at higher magnification (20X objective, lower row) for inflammatory cells. **Figure S8.** Splenocyte proliferation in response to different concentrations of *M. tuberculosis* restimulation. Splenocytes from a normal (preimmune) mouse and a CIA mouse were stimulated with 0 μg/ml 5μg/ml or 25 μg/ml *M. tuberculosis* extracts for 72 h. BrdU was added at the final 2 hours of stimulation. Proliferation (BrdU incorporation) of the CD45^+^ splenocytes was analyzed by flow cytometry.

**Additional file 2: **Supplementary methods.

## Data Availability

All data collected or analyzed in the study are presented in this published paper. Original data and experimental materials are available from the corresponding author upon reasonable request.

## Abbreviations

CTLA4: Cytotoxic T-lymphocyte protein 4
CTLA4Ig: CTLA4 and IgG Fc fusion protein
Alb-CTLA4Ig: Albumin-CTLA4Ig
MMP: Matrix metalloproteinase
ECD: Extracellular domain
CDR: Complementarity-determining region
EC_50_: Concentration that gives 50% of maximal response
IC_50_: Concentration that reduces 50% of maximal response
CIA: Collagen-induced arthritis
IL-2: Interleukin-2
TNF-α: Tumor necrosis factor-α
