# Supplementary material for: A lesion-selective albumin-CTLA4Ig as a safe and effective treatment for collagen-induced arthritis
Source: Inflamm Regen. 2023 Feb 16;43:13. doi: 10.1186/s41232-023-00264-8 (PMC9933273; doi:10.1186/s41232-023-00264-8)

Uncropped full-length western blot images used in **Figure 3**. Area enclosed in dashed rectangles are presented in the main text. Numbers on the sides are molecular weights.

Figure 3A

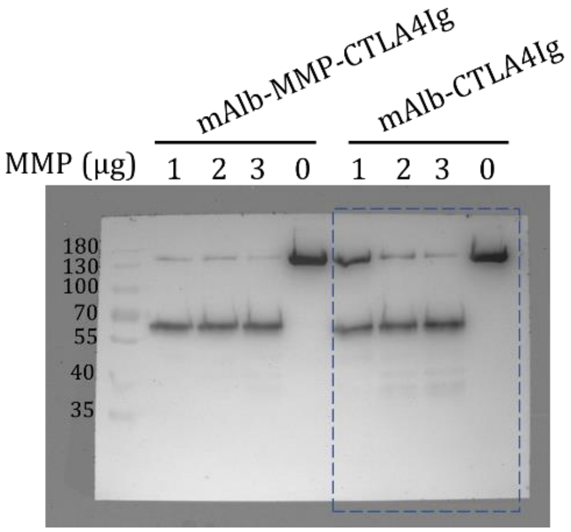

Figure 3C

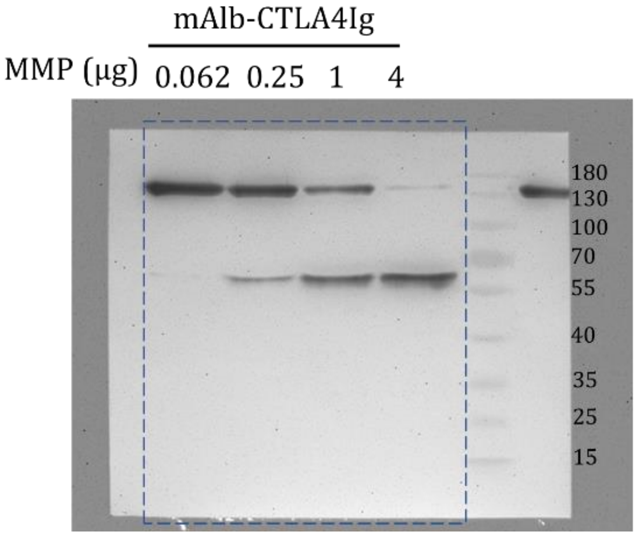

Supplement: Supplementary file 1 — Additional file 1: Figure S1. The N-terminal Ab lock and VpreB were unable to mask the binding activity of CTLA4Ig. (A) Binding activity of Ab lock-CTLA4Ig (0.5 μg/ml, blue line) and conventional CTLA4Ig (0.5 μg/ml, black line) to HEK-293 cells overexpressing CD80 (CD80 cells), detected by FITC-conjugated goat anti-mouse Fcγ in flow cytometry. Gray line: unstained cells. (B) Binding activity of VpreB-CTLA4Ig (0.5 μg/ml, blue line) and conventional CTLA4Ig (0.5 μg/ml, black line) to CD80 cells, detected by FITC-conjugated goat anti-mouse Fcγ antibodies by flow cytometry. Gray line: unstained cells. VpreB: immunoglobulin iota chain. (C) Simulation of Ab lock-mCTLA4Ig by the computer software BIOVIA Discovery Studio 2019 (Discovery Studio v19.1.0.18287). The structures of CTLA-4, the CDR3-like domain and the Ab lock are shown in magenta, yellow and light blue, respectively. Figure S2. Full recovery of the binding activity of mAlb-CTLA4Ig after MMP2/9 digestion. Nondigested, MMP-digested mAlb-CTLA44Ig, and conventional mCTLA4Ig (all at 1 nM) were added to the ELISA. Binding of the fusion proteins on the plate was detected by an HRP-conjugated anti-mouse IgG Fcγ secondary antibody. Figure S3. Characterization of an alternative Alb-CTLA4Ig with MMP substrate linker between albumin and CTLA4Ig (mAlb-MMP-CTLA4Ig). (A) Schematic representations of mAlb-MMP-CTLA4Ig constructs. MMP: MMP substrate sequence (GPLGMWSR) linker, eCTLA4: extracellular domain of CTLA4. P: promoter in the expression vector. (B) Reducing SDS-PAGE (left) and western blot analysis (right) of purified mAlb-MMP-CTLA4Ig. (C) The stability of mAlb-MMP-CTLA4Ig in DMEM containing 10% fetal bovine sera for seven days. (D) mAlb-MMP-CTLA4Ig were digested with the indicated amount of MMP2/9 and analyzed by western blot. (E) mAlb-MMP-CTLA4Ig were subjected to varying degrees of digestion by MMP2/9. Part of the digestion was analyzed by western blot to determine the degree of cleavage. The percent (%) cleaved Alb-MM [file 41232_2023_264_MOESM1_ESM.zip › Additional file 2_Uncropped image_Figure 3.pdf]
