# Supplementary material for: A lesion-selective albumin-CTLA4Ig as a safe and effective treatment for collagen-induced arthritis
Source: Inflamm Regen. 2023 Feb 16;43:13. doi: 10.1186/s41232-023-00264-8 (PMC9933273; doi:10.1186/s41232-023-00264-8)

Uncropped full-length SDS-PAGE and western blot images used in **Supplementary Figure 3.** Area enclosed in dashed rectangles are presented in the Supplementary Figure 3. Numbers on the sides are molecular weights.

Supplementary Figure 3B

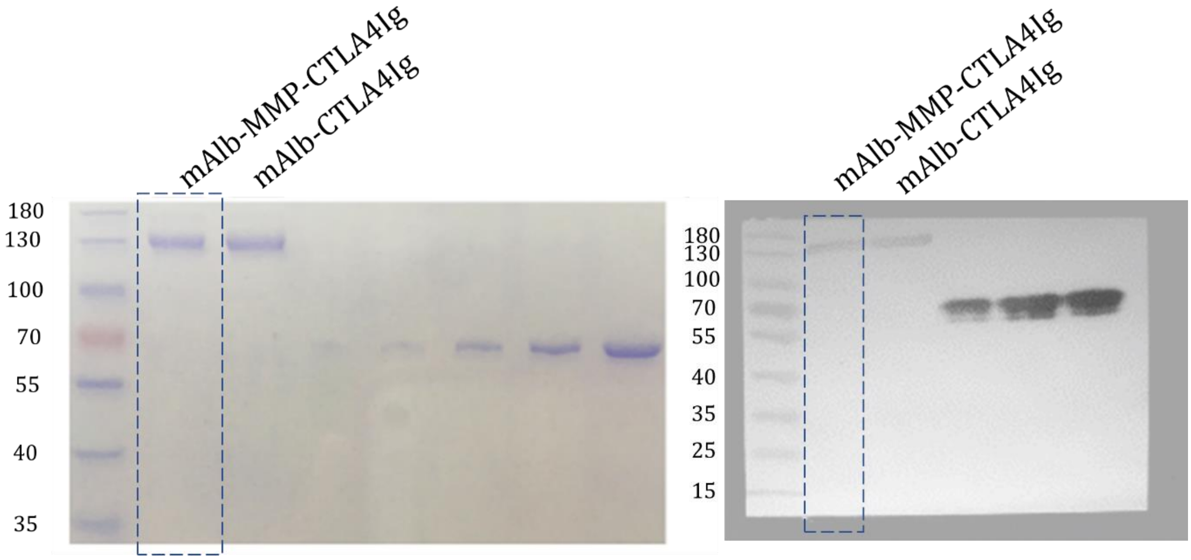

Supplementary Figure 3C

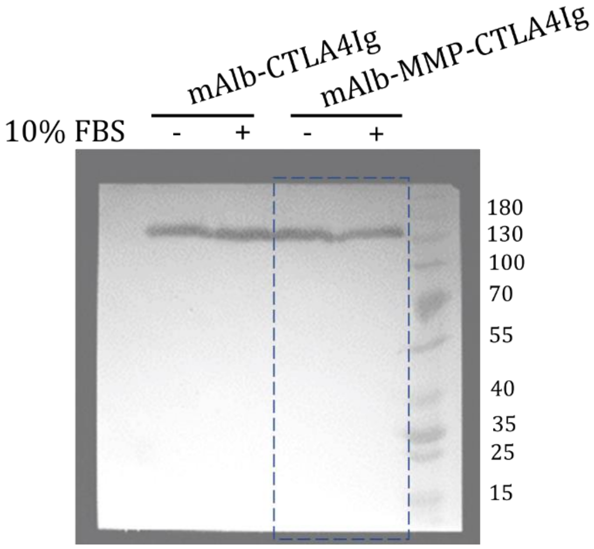

Supplementary Figure 3D

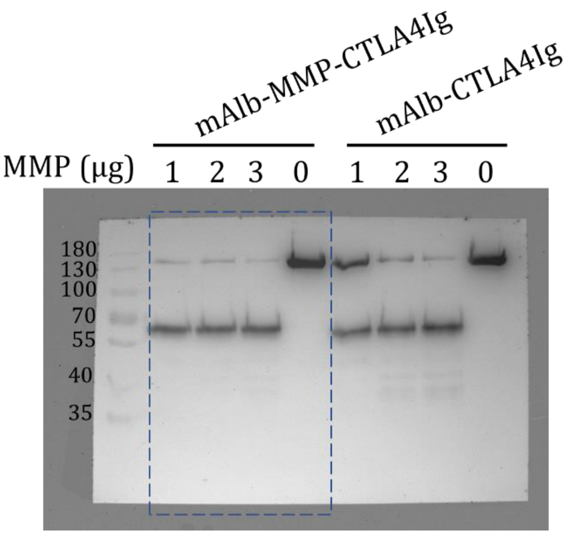

Supplementary Figure 3E

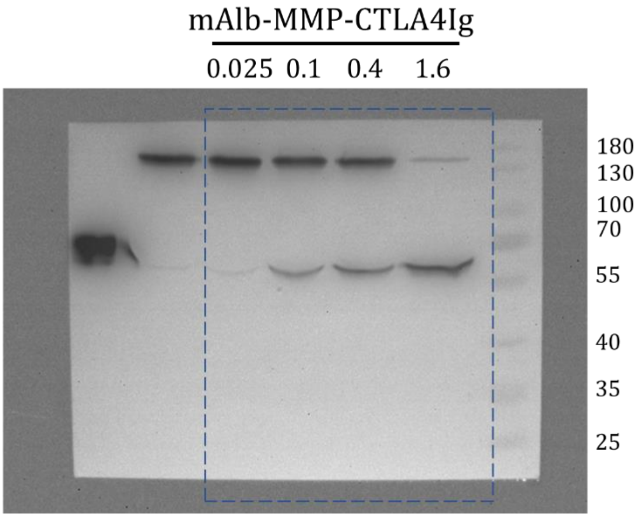

Supplement: Supplementary file 1 — Additional file 1: Figure S1. The N-terminal Ab lock and VpreB were unable to mask the binding activity of CTLA4Ig. (A) Binding activity of Ab lock-CTLA4Ig (0.5 μg/ml, blue line) and conventional CTLA4Ig (0.5 μg/ml, black line) to HEK-293 cells overexpressing CD80 (CD80 cells), detected by FITC-conjugated goat anti-mouse Fcγ in flow cytometry. Gray line: unstained cells. (B) Binding activity of VpreB-CTLA4Ig (0.5 μg/ml, blue line) and conventional CTLA4Ig (0.5 μg/ml, black line) to CD80 cells, detected by FITC-conjugated goat anti-mouse Fcγ antibodies by flow cytometry. Gray line: unstained cells. VpreB: immunoglobulin iota chain. (C) Simulation of Ab lock-mCTLA4Ig by the computer software BIOVIA Discovery Studio 2019 (Discovery Studio v19.1.0.18287). The structures of CTLA-4, the CDR3-like domain and the Ab lock are shown in magenta, yellow and light blue, respectively. Figure S2. Full recovery of the binding activity of mAlb-CTLA4Ig after MMP2/9 digestion. Nondigested, MMP-digested mAlb-CTLA44Ig, and conventional mCTLA4Ig (all at 1 nM) were added to the ELISA. Binding of the fusion proteins on the plate was detected by an HRP-conjugated anti-mouse IgG Fcγ secondary antibody. Figure S3. Characterization of an alternative Alb-CTLA4Ig with MMP substrate linker between albumin and CTLA4Ig (mAlb-MMP-CTLA4Ig). (A) Schematic representations of mAlb-MMP-CTLA4Ig constructs. MMP: MMP substrate sequence (GPLGMWSR) linker, eCTLA4: extracellular domain of CTLA4. P: promoter in the expression vector. (B) Reducing SDS-PAGE (left) and western blot analysis (right) of purified mAlb-MMP-CTLA4Ig. (C) The stability of mAlb-MMP-CTLA4Ig in DMEM containing 10% fetal bovine sera for seven days. (D) mAlb-MMP-CTLA4Ig were digested with the indicated amount of MMP2/9 and analyzed by western blot. (E) mAlb-MMP-CTLA4Ig were subjected to varying degrees of digestion by MMP2/9. Part of the digestion was analyzed by western blot to determine the degree of cleavage. The percent (%) cleaved Alb-MM [file 41232_2023_264_MOESM1_ESM.zip › Additional file 5_uncropped image_SuppFigure 3.pdf]
