# Supplementary material for: A lesion-selective albumin-CTLA4Ig as a safe and effective treatment for collagen-induced arthritis
Source: Inflamm Regen. 2023 Feb 16;43:13. doi: 10.1186/s41232-023-00264-8 (PMC9933273; doi:10.1186/s41232-023-00264-8)
Supplement: Supplementary file 1 — Additional file 1: Figure S1. The N-terminal Ab lock and VpreB were unable to mask the binding activity of CTLA4Ig. (A) Binding activity of Ab lock-CTLA4Ig (0.5 μg/ml, blue line) and conventional CTLA4Ig (0.5 μg/ml, black line) to HEK-293 cells overexpressing CD80 (CD80 cells), detected by FITC-conjugated goat anti-mouse Fcγ in flow cytometry. Gray line: unstained cells. (B) Binding activity of VpreB-CTLA4Ig (0.5 μg/ml, blue line) and conventional CTLA4Ig (0.5 μg/ml, black line) to CD80 cells, detected by FITC-conjugated goat anti-mouse Fcγ antibodies by flow cytometry. Gray line: unstained cells. VpreB: immunoglobulin iota chain. (C) Simulation of Ab lock-mCTLA4Ig by the computer software BIOVIA Discovery Studio 2019 (Discovery Studio v19.1.0.18287). The structures of CTLA-4, the CDR3-like domain and the Ab lock are shown in magenta, yellow and light blue, respectively. Figure S2. Full recovery of the binding activity of mAlb-CTLA4Ig after MMP2/9 digestion. Nondigested, MMP-digested mAlb-CTLA44Ig, and conventional mCTLA4Ig (all at 1 nM) were added to the ELISA. Binding of the fusion proteins on the plate was detected by an HRP-conjugated anti-mouse IgG Fcγ secondary antibody. Figure S3. Characterization of an alternative Alb-CTLA4Ig with MMP substrate linker between albumin and CTLA4Ig (mAlb-MMP-CTLA4Ig). (A) Schematic representations of mAlb-MMP-CTLA4Ig constructs. MMP: MMP substrate sequence (GPLGMWSR) linker, eCTLA4: extracellular domain of CTLA4. P: promoter in the expression vector. (B) Reducing SDS-PAGE (left) and western blot analysis (right) of purified mAlb-MMP-CTLA4Ig. (C) The stability of mAlb-MMP-CTLA4Ig in DMEM containing 10% fetal bovine sera for seven days. (D) mAlb-MMP-CTLA4Ig were digested with the indicated amount of MMP2/9 and analyzed by western blot. (E) mAlb-MMP-CTLA4Ig were subjected to varying degrees of digestion by MMP2/9. Part of the digestion was analyzed by western blot to determine the degree of cleavage. The percent (%) cleaved Alb-MM [file 41232_2023_264_MOESM1_ESM.zip › Supplementary Figures and Legends_ESM.docx]

Figure S1


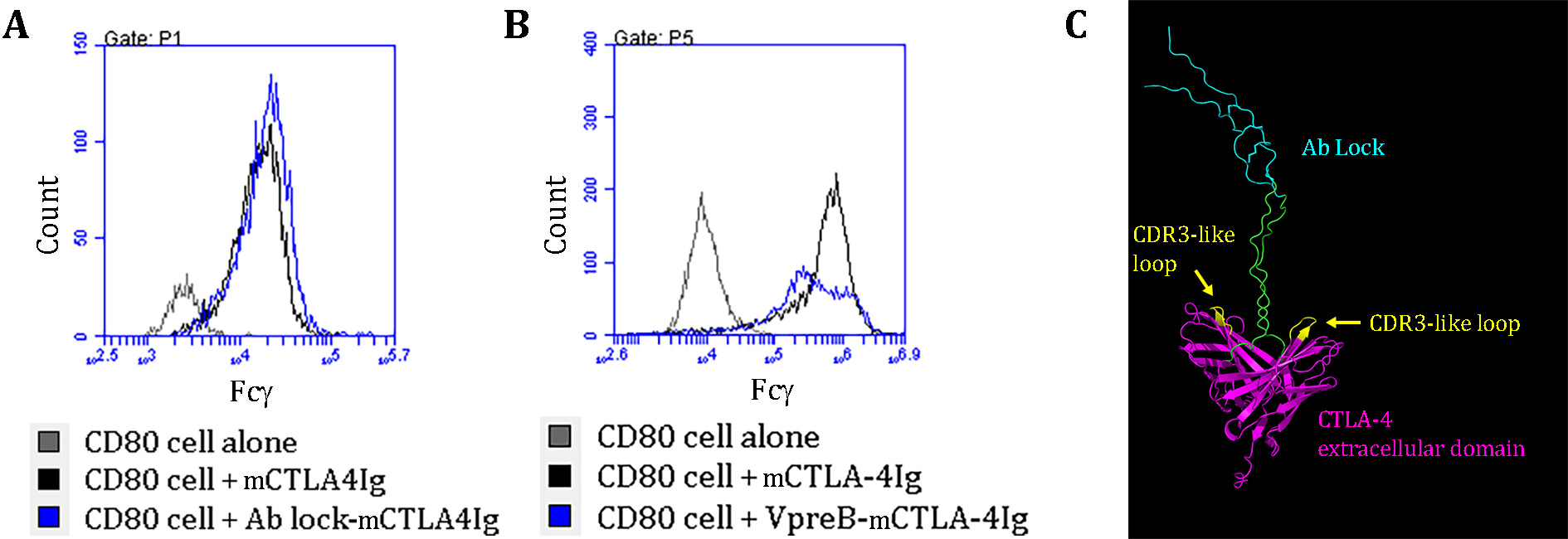


**Figure S1. The N-terminal Ab lock and VpreB were unable to mask the binding activity of CTLA4Ig. (A)** Binding activity of Ab lock-CTLA4Ig (0.5 μg/ml, blue line) and conventional CTLA4Ig (0.5 μg/ml, black line) to HEK-293 cells overexpressing CD80 (CD80 cells), detected by FITC-conjugated goat anti-mouse Fcγ in flow cytometry. Gray line: unstained cells. **(B)** Binding activity of VpreB-CTLA4Ig (0.5 µg/ml, blue line) and conventional CTLA4Ig (0.5 µg/ml, black line) to CD80 cells, detected by FITC-conjugated goat anti-mouse Fcγ antibodies by flow cytometry. Gray line: unstained cells. VpreB: immunoglobulin iota chain. **(C)** Simulation of Ab lock-mCTLA4Ig by the computer software BIOVIA Discovery Studio 2019 ((Discovery Studio v19.1.0.18287). The structures of CTLA-4, the CDR3-like domain and the Ab lock are shown in magenta, yellow and light blue, respectively.

Figure S2

**Figure S2. Full recovery of the binding activity of mAlb-CTLA4Ig after MMP2/9 digestion.** Nondigested, MMP-digested mAlb-CTLA44Ig, and conventional mCTLA4Ig (all at 1 nM) were added to the ELISA. Binding of the fusion proteins on the plate was detected by an HRP-conjugated anti-mouse IgG Fcγ secondary antibody.


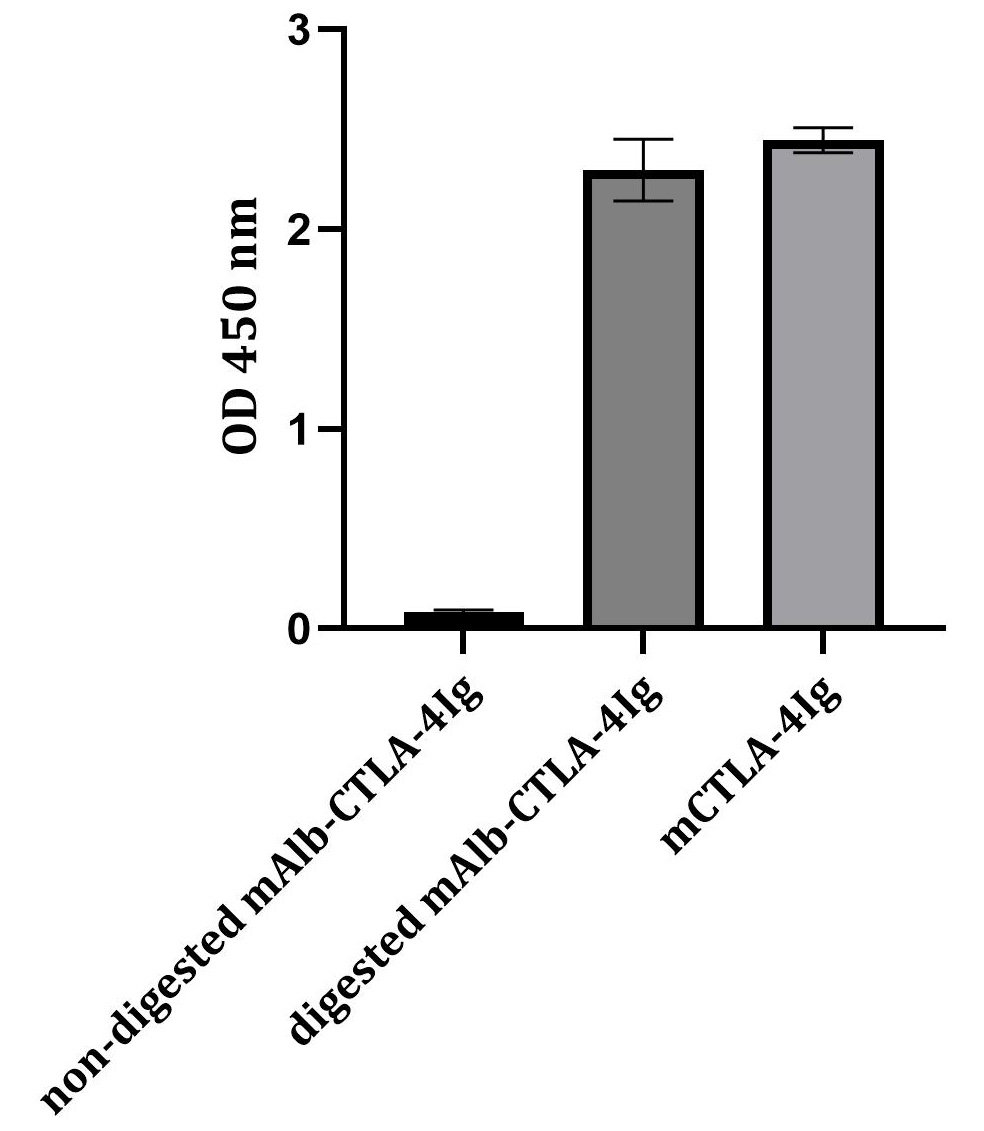


Figure S3


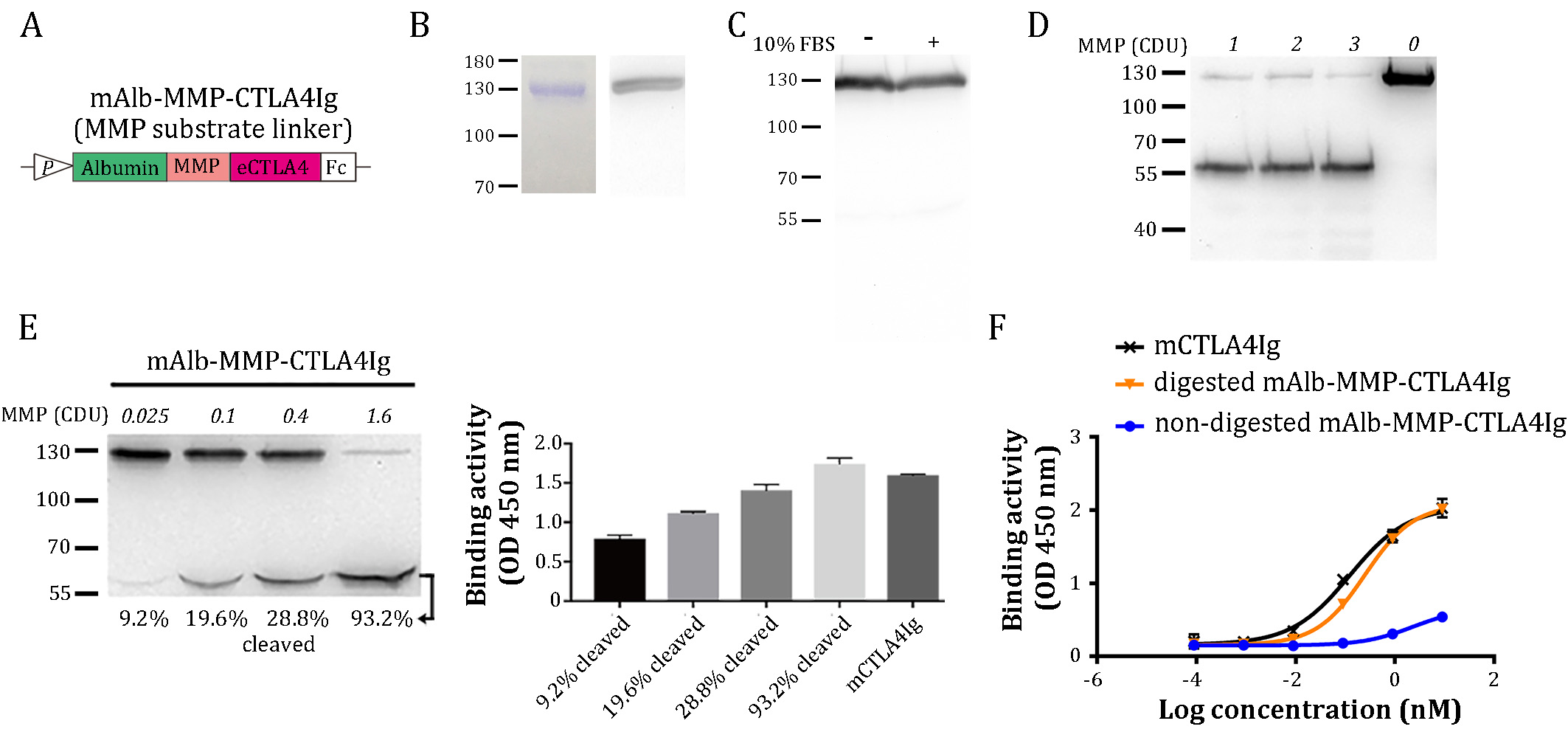


**Figure S3. Characterization of an alternative Alb-CTLA4Ig with MMP substrate linker between albumin and CTLA4Ig (mAlb-MMP-CTLA4Ig). (A)** Schematic representations of mAlb-MMP-CTLA4Ig constructs. MMP: MMP substrate sequence (GPLGMWSR) linker, eCTLA4: extracellular domain of CTLA4. P: promoter in the expression vector. **(B)** Reducing SDS-PAGE (left) and western blot analysis (right) of purified mAlb-MMP-CTLA4Ig. **(C)** The stability of mAlb-MMP-CTLA4Ig in DMEM containing 10% fetal bovine sera for seven days. **(D)** mAlb-MMP-CTLA4Ig were digested with the indicated amount of MMP2/9 and analyzed by western blot. **(E)** mAlb-MMP-CTLA4Ig were subjected to varying degrees of digestion by MMP2/9. Part of the digestion was analyzed by western blot to determine the degree of cleavage. The percent (%) cleaved Alb-MMP-CTLA4Ig was quantitated and is indicated below each lane. The digestion was added to the cell-based ELISA. The binding of CD80 by 1 nM conventional mCTLA4Ig was used as a positive control. **(F)** Binding kinetics of mAlb-MMP-CTLA4Ig before (blue curve) and after MMP digestion (orange curve). The binding kinetics of conventional mCTLA4Ig are shown in the black curve.

Figure S4


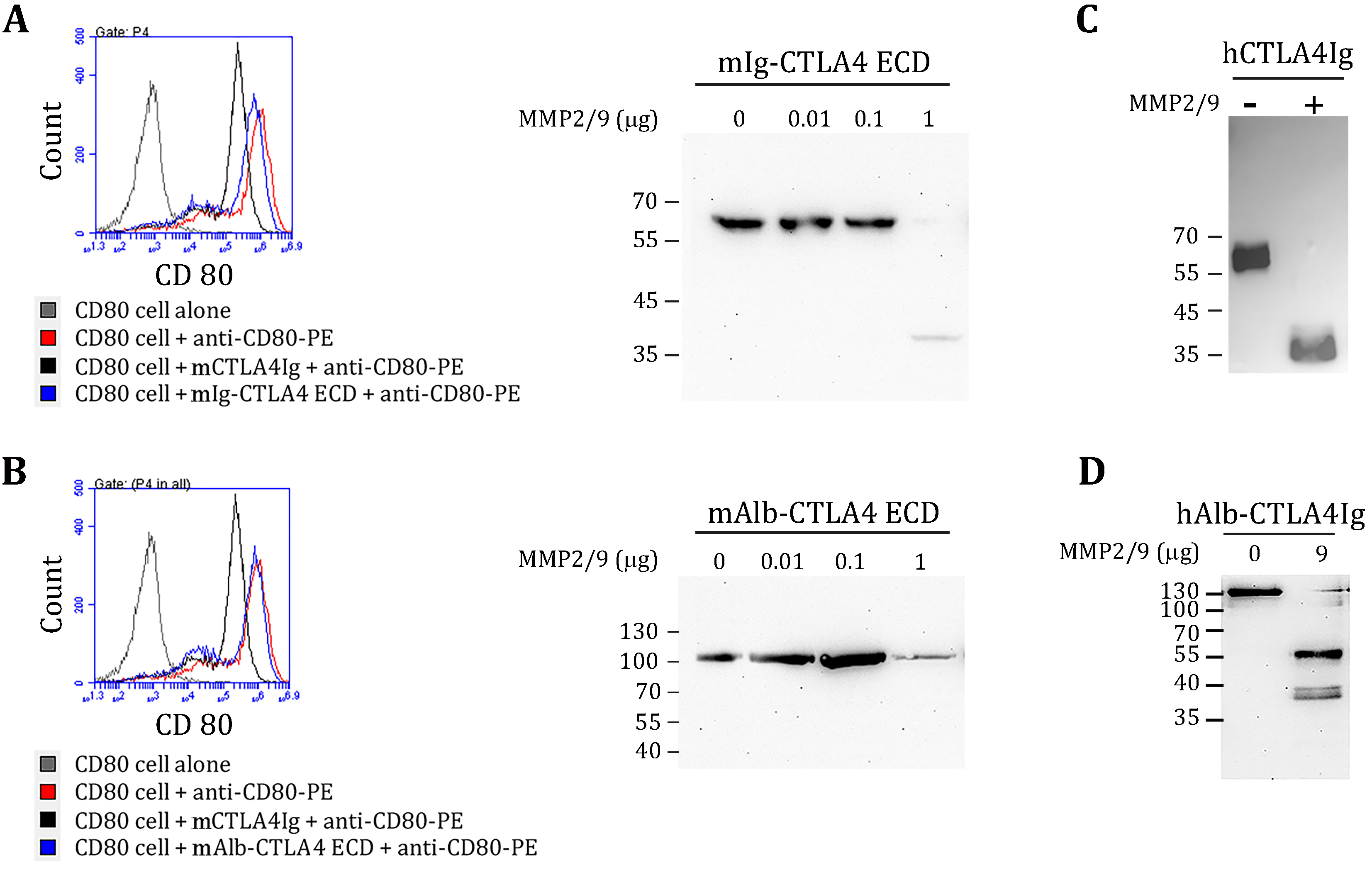


**Figure S4. Lacking an N-terminal albumin and a C-terminal Fc decreases masking efficiency and stability. (A)** Competitive binding of Ig-CTLA4 ECD (0.5 μg/ml, blue line) or conventional CTLA4Ig (0.5 μg/ml, black line) against PE-conjugated anti-mouse CD80 antibodies (1 μg/ml) to HEK-293 cells overexpressing CD80 (CD80 cells). Red line: CD80-expressing cells stained with PE-conjugated anti-CD 80 antibodies alone (1 µg/ml). Gray line: unstained cells. Digestion products of the IgG1 Fc-CTLA4 ECD by the indicated amounts of MMP2/9 are shown in the western blot using anti-mouse Fcγ antibodies (right panel). **(B)** Competitive binding activity of Alb-CTLA4 ECD (0.5 μg/ml, blue line) and conventional CTLA4Ig (0.5 μg/ml, black line) to CD80 cells in the presence of PE-conjugated anti-mouse CD80 antibodies (1 μg/ml) by flow cytometry. Red line: CD80 cells stained with PE-conjugated anti-CD80 antibody alone (1 µg/ml). Gray line: unstained cells. Digestion products of the Alb-CTLA4 ECD by the indicated amounts of MMP2/9 are shown in the western blot using anti-mouse CTLA4 antibodies (right panel). **(C)** Digestion products of conventional human CTLA4Ig (3.6 picomole) by 2 units of MMP2/9 is shown in the western blot using anti-human Fcγ antibodies. **(D)** Overdigestion of hAlb-CTLA4Ig (1.35 picomole) in higher amounts (9 units) of MMP2/9.

Figure S5


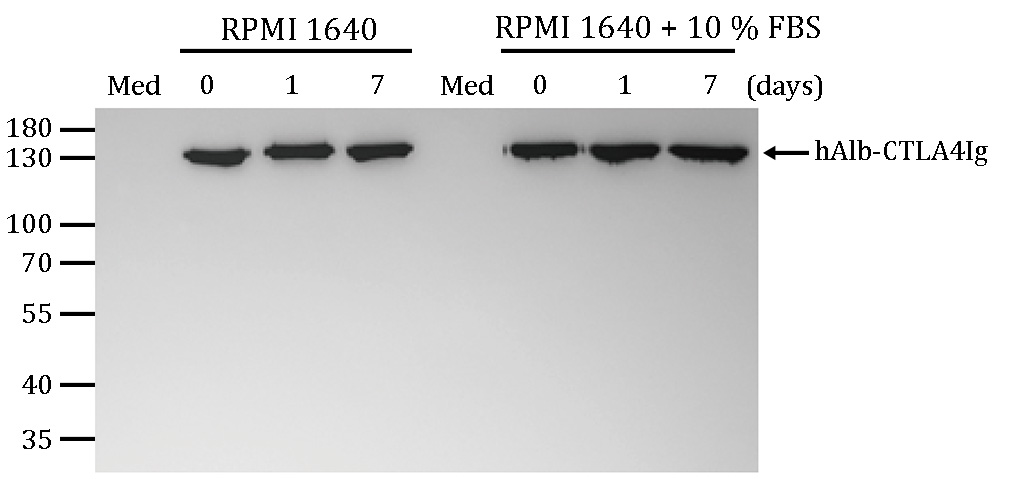


**Figure S5. The stability of hAlb-CTLA4Ig in sera**. hAlb-CTLA4Ig incubated in RPMI 1640 containing 10% fetal bovine sera for seven days was analyzed by western blot using an anti-human Fcγ secondary antibody. Med: medium alone.

Figure S6


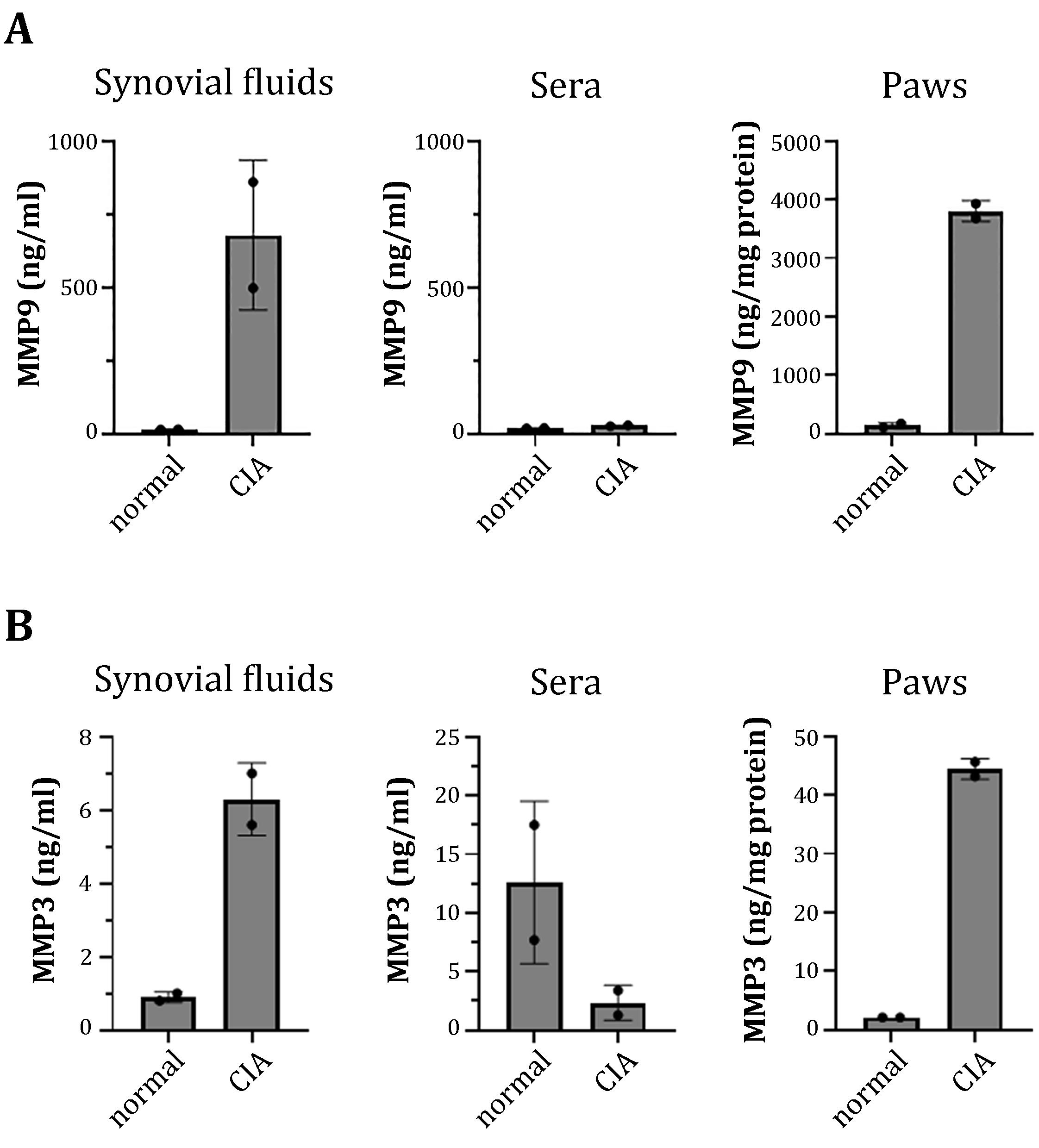


**Figure S6. Differential levels of MMP9 and MMP3 in normal control mice and CIA mice.** Protein levels of MMP9 **(A)** and MMP3 **(B)** in the synovial fluid lavages, sera, and paws of normal control mice and CIA mice were measured by ELISA. Protein levels are expressed as ng/ml in synovial fluid lavages and sera, ng/mg protein in protein extracts of the paws.

Figure S7

**
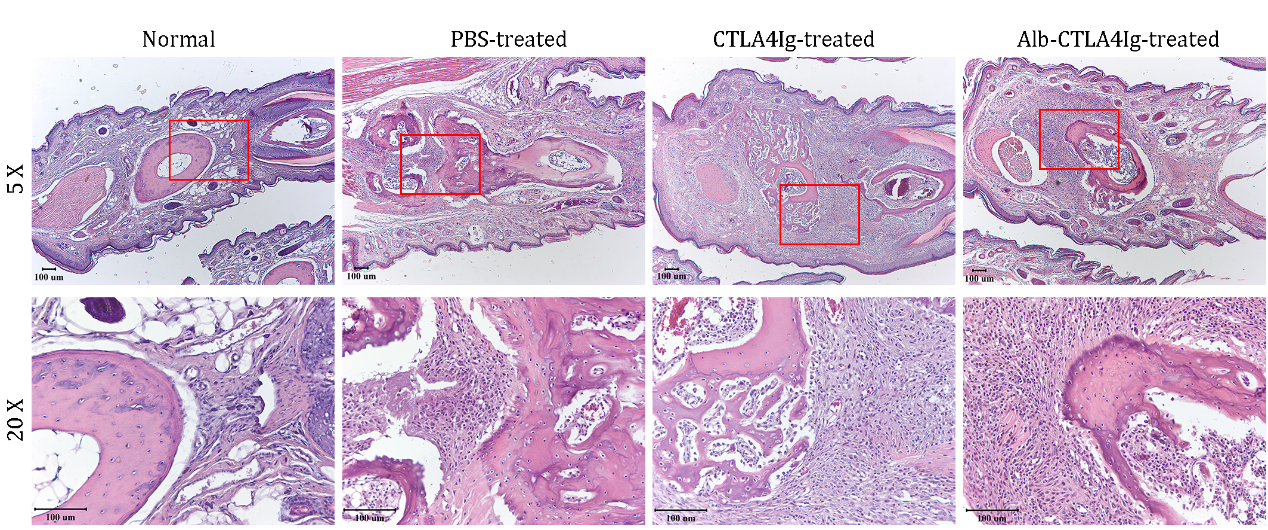
**

**Figure S7. Histopathological microphotographs of the digits of a normal mouse or CIA mice.** The area enclosed by red rectangles at low magnification (5X objective, upper row) is shown at higher magnification (20X objective, lower row) for inflammatory cells.

Figure S8


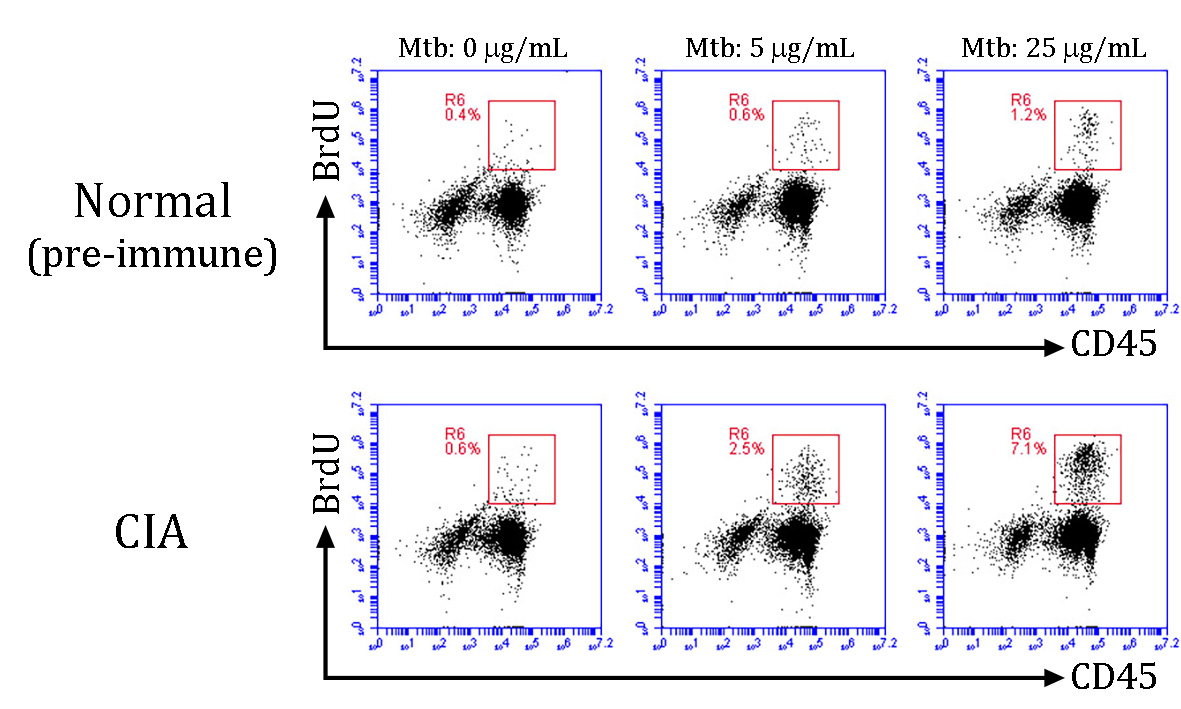


**Figure S8. Splenocyte proliferation in response to different concentrations of *M. tuberculosis* restimulation**. Splenocytes from a normal (preimmune) mouse and a CIA mouse were stimulated with 0 μg/ml 5μg/ml or 25 μg/ml *M. tuberculosis* extracts for 72 h. BrdU was added at the final 2 hours of stimulation. Proliferation (BrdU incorporation) of the CD45^+^ splenocytes was analyzed by flow cytometry.
