# Supplementary material for: A lesion-selective albumin-CTLA4Ig as a safe and effective treatment for collagen-induced arthritis
Source: Inflamm Regen. 2023 Feb 16;43:13. doi: 10.1186/s41232-023-00264-8 (PMC9933273; doi:10.1186/s41232-023-00264-8)
Supplement: Supplementary file 2 — Additional file 2: Supplementary methods. [file 41232_2023_264_MOESM2_ESM.docx]

**Supplementary Methods**

*Construction of CTLA4Ig and Alb-CTLA4Ig expression vectors*

The coding sequences of mouse albumin (Alb) and the extracellular domain of mouse CTLA4 were reverse-transcribed from mRNA by Superscript III (Thermo Fisher Scientific, Waltham, MA, USA) using tissue lysates prepared from a mouse liver and a mouse spleen with TRIzol reagent (Thermo Fischer Scientific). The coding sequences of human albumin and the extracellular domain of human CTLA4 were custom-synthesized (Genomics, New Taipei, Taiwan). To construct mouse or human CTLA4Ig, we linked the coding sequences of the mouse or human extracellular domain of CTLA4 (CTLA4 ECD) to the coding sequences of Fc fragments of mouse IgG_2a_ or human IgG_1_ by overlapping PCR (1). To construct Alb-CTLA4Ig, we first linked the coding sequences of albumin and the CTLA4 ECD through MMP-cleavable sequences (mouse MMP linkers: GPLGMWSR (1), mouse lower hinge sequence: CPPCKCPAPNLLGGP (2); human MMP linkers: GPLGVR (3), human lower hinge sequence: CPPCPAPELLGGP (4)) by overlapping PCR. The Alb-CTLA4 ECD sequences were then linked to the coding sequences of mouse or human Ig (Fc fragment of IgG) by a second overlapping PCR to derive mouse or human Alb-CTLA4Ig. A 6X-histidine tag (His-tag) was added to the 3'-end of the fusion constructs during the second overlapping PCR to aid downstream purification of the recombinant proteins by immobilized nickel affinity chromatography. The DNA fragments of mouse or human Alb-CTLA4Ig were subcloned into pLNCX vectors (1) for mammalian expression of the fusion proteins. The mouse or human Alb-CTLA4Ig constructs were sequenced to ensure no mutation in coding sequences and an accurate reading frame. Table S1 below summarizes the starting/ending amino acids for each protein domain in the constructs.


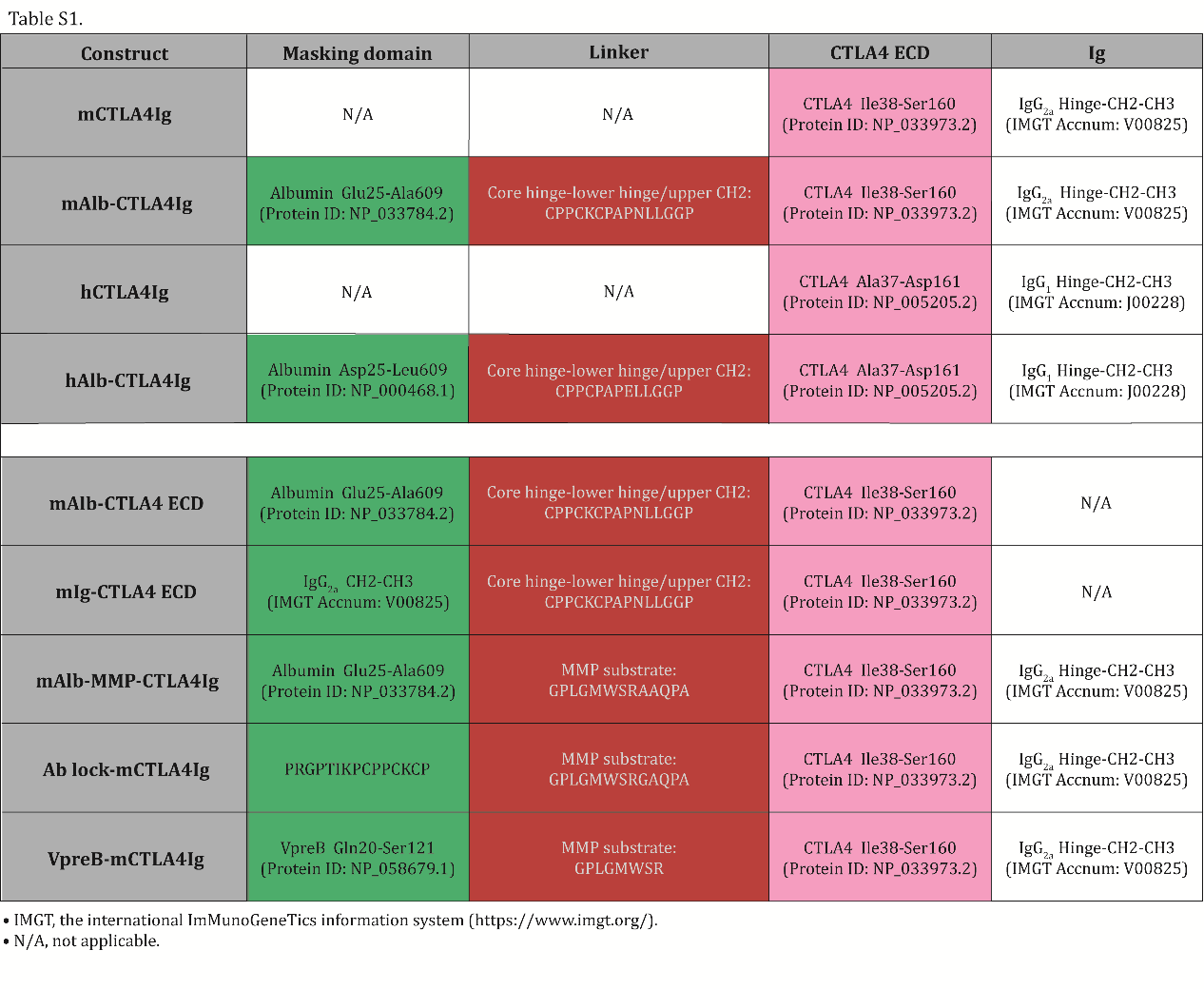


*Generation of HEK 293 cells overexpressing CD80*

Coding sequences of full-length mouse and human CD80 were cloned from spleen lysates or custom-synthesized and then subcloned into the pLNCX vectors. HEK-293 cells were transfected with mouse or human CD80 expression vectors using TransIT transfection reagents (Mirus Bio, Madison, WI, USA). HEK-293 transfectants were selected for stable expression of the vector by 500 μg/ml G418 (Thermo Fisher Scientific). Cells that expressed high levels of mouse or human CD80 were sorted by a FACSAria flow cytometer (BD Bioscience, San Jose, CA, USA).

*Detection of MMPs by ELISA*

To collect synovial fluid lavages as much as possible, we injected 30 μl (10 μl for 3 times) of DMEM into the synovial cavity of each knee joint of normal mice (N=2) and CIA mice (N=2). A total of ~60 μl of synovial fluid lavages was obtained for each mouse. We also collected the sera and paws of normal control mice and CIA mice, and homogenized the paws in PBS (0.5 ml) using a pestle and mortar with liquid nitrogen. Synovial fluid lavages were diluted 10-fold and added to the MMP9 or MMP3 ELISA plates (Elabscience, Houston, TX, USA). Sera were 10-, 100-, and 1000-fold diluted and added to the ELISA plates. Protein extractions of the paws were quantified by a BCA assay, then 2, 10, 50 μg of protein extracts were added to the ELISA plates. The levels of MMP9 and MMP3 in the synovial fluid lavages, sera, and paws were detected using the reagents and protocols provided by the manufacturer. MMP9 and MMP3 levels were calculated by adjusting the dilution fold or protein concentration, expressed as ng/ml in the synovial lavages and sera, and ng/mg protein extracts in the paws.

*Flow cytometry of synovium-infiltrating cells*

Cells in the synovial fluids were isolated after injection of DMEM into the knee joints of mice that developed CIA. Synovium membranes covering the knee joints of CIA mice were dissected, cut into small pieces, and digested in type I collagenase (3 mg/ml) (Sigma-Aldrich). Cells in the synovial fluid lavage or collagenase digestion were collected after centrifugation. The cells were washed and blocked for nonspecific antibody binding by TruStain FcX (BioLegend) and then stained with fluorochrome-conjugated antibodies against CD3, CD45, B220, Gr-1, or Ly-6G (BioLegend) on ice for 30 min. The cells were washed and fixed with 2% paraformaldehyde in PBS and analyzed by a flow cytometer (C6, BD Bioscience).

In vitro *effects of mCTLA4Ig on synovium-infiltrating cells*

Plate-bound immunocomplex was used to activate synovium-infiltrating cells (primarily neutrophils). In brief, sterile bovine type II collagen (50 μg/ml in 0.1 M phosphate buffer, 50 μl per well) was coated on the 96-well plate at 4 °C overnight. The plates were washed in sterile PBS, and CIA or normal serum (1:1000 diluted in PBS, 50 μl per well) was added at room temperature for 2 h and then washed in PBS. Synovium-infiltrating cells were isolated from the knee joints of CIA mice, washed, added to the plates (200,000 cells/well) in culture media (DMEM supplemented with 10% FBS, 1% penicillin and streptomycin), and treated with CTLA4Ig (1 and 5 μg/ml) or isotype control (1 μg/ml) for 24 h. The culture supernatants were collected, and the concentrations of tumor necrosis factor-α (TNF-α) were measured using an ELISA kit (R & D Systems, Minneapolis, MN, USA).

*Extraction of* M. tuberculosis *water-soluble antigens*

To prepare microbial antigens, 30 mg of M. tuberculosis was first ground by a mortar and pestle, added to 0.5 ml PBS, and snap-frozen in liquid nitrogen, followed by repeated grinding twice. The total extracts were centrifuged at 1000 × g for 10 min, and liquid extracts in the supernatants were transferred and sterilized by passing the extracts through a 0.22 μm syringe filter. The extracts were quantified by a BCA kit (Thermo Fisher Scientific) and stored at -80 °C until use.

**References**

1. Lee CJ, Wang CC, Chen M, Chuang KH, Cheng TL, Jian TY, et al. Development of an inflammatory tissue-selective chimeric TNF receptor. Cytokine. 2019;113:340-6.

2. Gearing AJ, Thorpe SJ, Miller K, Mangan M, Varley PG, Dudgeon T, et al. Selective cleavage of human IgG by the matrix metalloproteinases, matrilysin and stromelysin. Immunology letters. 2002;81(1):41-8.

3. Chuang CH, Chuang KH, Wang HE, Roffler SR, Shiea JT, Tzou SC, et al. In vivo positron emission tomography imaging of protease activity by generation of a hydrophobic product from a noninhibitory protease substrate. Clinical cancer research : an official journal of the American Association for Cancer Research. 2012;18(1):238-47.

4. Brezski RJ, Jordan RE. Cleavage of IgGs by proteases associated with invasive diseases: an evasion tactic against host immunity? mAbs. 2010;2(3):212-20.
